# Insulin and Insulin-Like Growth Factor 1 Signaling Preserves Sarcomere Integrity in the Adult Heart

**DOI:** 10.1128/mcb.00163-22

**Published:** 2022-09-20

**Authors:** Christian Riehle, Eric T. Weatherford, Nicholas S. McCarty, Alec Seei, Bharat P. Jaishy, Rajkumar Manivel, Paolo Galuppo, Chantal Allamargot, Tariq Hameed, Ryan L. Boudreau, Johann Bauersachs, Robert M. Weiss, E. Dale Abel

**Affiliations:** a Fraternal Order of Eagles Diabetes Research Center, University of Iowa, Iowa City, Iowa, USA; b Division of Endocrinology and Metabolism, Carver College of Medicine, University of Iowa, Iowa City, Iowa, USA; c Department of Cardiology and Angiology, Hannover Medical School, Hannover, Germany; d Central Microscopy Research Facility, Carver College of Medicine, University of Iowa, Iowa City, Iowa, USA; e Division of Cardiovascular Medicine, Carver College of Medicine, University of Iowa, Iowa City, Iowa, USA; f Department of Internal Medicine, Carver College of Medicine, University of Iowa, Iowa City, Iowa, USA

**Keywords:** heart failure, cardiac remodeling, insulin signaling, cardiac structure

## Abstract

Insulin and insulin-like growth factor 1 (IGF1) signaling is transduced by insulin receptor substrate 1 (IRS1) and IRS2. To elucidate physiological and redundant roles of insulin and IGF1 signaling in adult hearts, we generated mice with inducible cardiomyocyte-specific deletion of insulin and IGF1 receptors or IRS1 and IRS2. Both models developed dilated cardiomyopathy, and most mice died by 8 weeks post-gene deletion. Heart failure was characterized by cardiomyocyte loss and disarray, increased proapoptotic signaling, and increased autophagy. Suppression of autophagy by activating mTOR signaling did not prevent heart failure. Transcriptional profiling revealed reduced serum response factor (SRF) transcriptional activity and decreased mRNA levels of genes encoding sarcomere and gap junction proteins as early as 3 days post-gene deletion, in concert with ultrastructural evidence of sarcomere disruption and intercalated discs within 1 week after gene deletion. These data confirm conserved roles for constitutive insulin and IGF1 signaling in suppressing autophagic and apoptotic signaling in the adult heart. The present study also identifies an unexpected role for insulin and IGF1 signaling in regulating an SRF-mediated transcriptional program, which maintains expression of genes encoding proteins that support sarcomere integrity in the adult heart, reduction of which results in rapid development of heart failure.

## INTRODUCTION

Insulin receptors (IR) and insulin-like growth factor 1 (IGF1) receptors (IGF1R) are closely related receptors that play important roles in cardiac growth and metabolism following activation by their cognate ligands. IR and IGF1R are structurally similar α_2_β_2_ heterotetrameric transmembrane receptors consisting of ligand-binding transmembrane α-subunits and intracellular β-subunits, which exhibit tyrosine kinase activity. IR and IGF1R may exist in the heart as heterodimers that respond to both ligands, leading to redundant and overlapping functions (1). Although IR and IGF1R share common interaction partners, i.e., insulin receptor substrates (IRS), IR and IGF1R play distinct roles in cardiac development and size. Previous reports demonstrated that constitutive cardiomyocyte-specific deletion of the IGF1R during prenatal development had no effect on postnatal cardiac size but attenuated the hypertrophic response to endurance exercise training (2). In contrast, prenatal knockout of the IR in cardiomyocytes (CIRKO) reduced postnatal cardiac size by 20 to 30% (3). CIRKO hearts demonstrated increased glycolysis, decreased fatty acid oxidation rates (3), and attenuated maturation of cardiac mitochondrial oxidative capacity (4). However, isolated deficiency of the IR or the IGF1R was well tolerated and did not impact survival in the absence of hemodynamic stress (5–7). The relatively mild phenotype observed in these models suggests that IR or IGF1R signaling is sufficient to preserve contractile function in the nonstressed heart consistent with functional redundancy. This conclusion is also supported by a mouse model with combined perinatal cardiomyocyte-specific deletion of both IR and IGF1R that exhibited fatal cardiomyopathy and died by the age of 4 weeks (8).

Studies using mice with germline and cardiomyocyte-specific deletion of IRS isoforms suggest a predominant role for IRS1 in the regulation of growth and for IRS2 in the regulation of metabolism (9–12). In addition, mice with cardiomyocyte-specific knockout of IRS1 or IRS2 have preserved contractile function at baseline (12). In contrast, combined perinatal knockout of IRS1 and IRS2 in the heart resulted in dilated cardiomyopathy (DCM) and premature death and exhibited excessive autophagy and mitochondrial dysfunction (13–15). The present study sought to determine the physiological role of these redundant signaling pathways in the adult heart by acutely impairing the ability of the adult heart to respond to these growth factors by inducible deletion of IR/IGF1R or IRS1/2. We generated mice with combined inducible deletion of IR/IGF1R (iCIR^2^KO) or IRS1/2 (iCIRS12KO). We observed increased autophagy in both models, which was attenuated by activation of mTOR signaling by supplementation with branched-chain amino acids (BCAA). However, in contrast to the beneficial effect of autophagy reduction that we previously observed in mice with prenatal loss of IRS1/2 (13), autophagy suppression did not attenuate heart failure in these models. These data reveal the existence of additional insulin-responsive targets that maintain myocardial structure and function. Here, we identify a role for IR/IGF1R-mediated signaling in regulating a serum response factor (SRF)-mediated transcriptional program necessary for sarcomeric integrity in the adult heart, which if absent results in rapid development of heart failure.

## RESULTS

### Impaired insulin-mediated signaling and heart failure in iCIR^2^KO and iCIRS12KO hearts.

**FIG 1:**
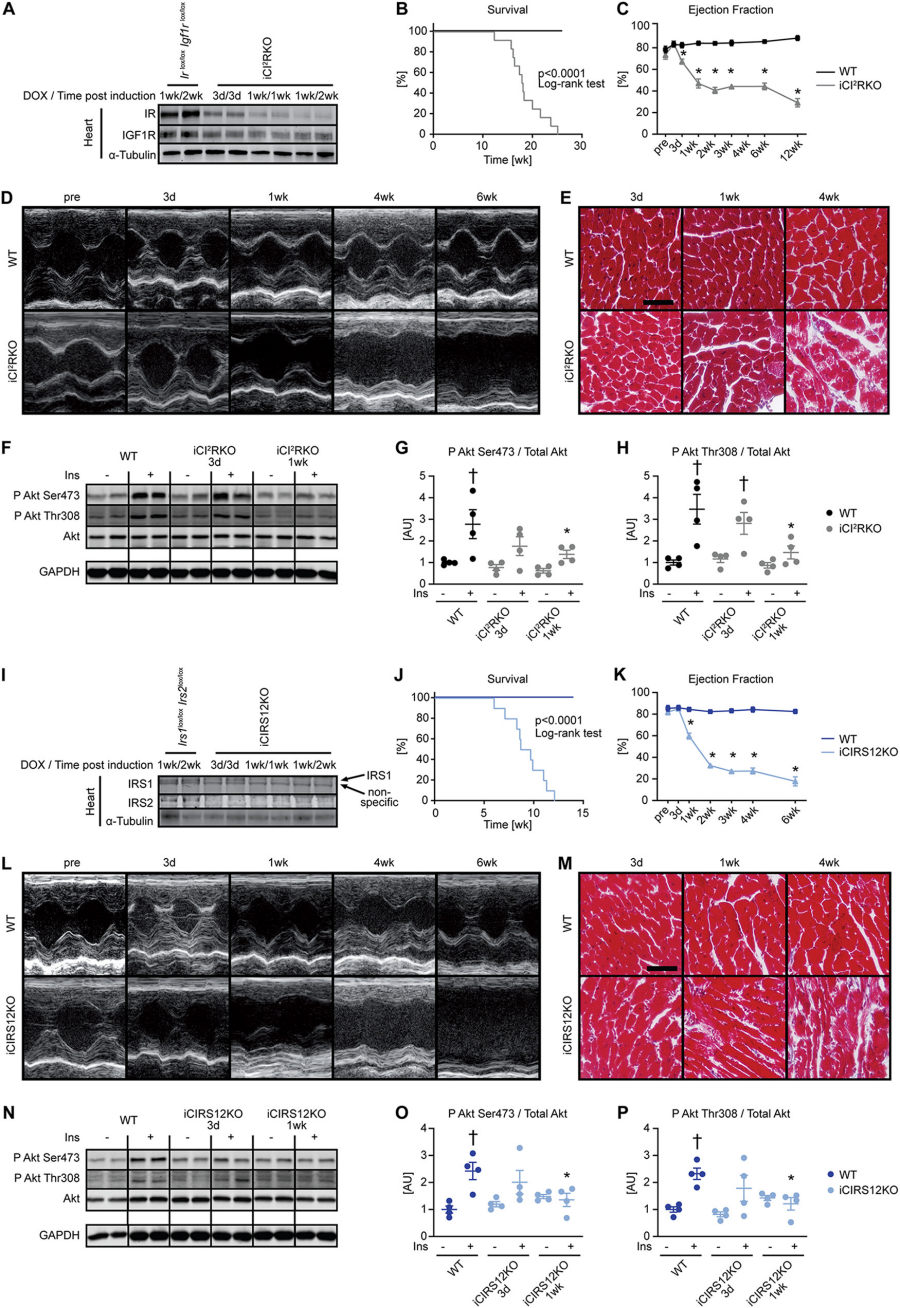
Impaired insulin-mediated signaling and heart failure in iCIR^2^KO and iCIRS12KO hearts. Two-way ANOVA was performed to analyze differences after insulin stimulation by genotype, followed by Newman-Keuls *post hoc* analysis (#, *P* < 0.05 for insulin stimulation; $, *P* < 0.05 for genotype; &, *P* < 0.05 for the interaction between insulin stimulation and genotype; these symbol definitions apply to symbols used not in the figure itself but in the panel descriptions below). (A) Representative immunoblots for IR and IGF1R in ventricle homogenates from mice with genotypes at time points as indicated. DOX, doxycycline. (B) Survival curves (*n* = 11 or 12). (C to E) Time course for ejection fraction (*n* = 7 or 8) (C), representative M-mode images (D), and representative trichrome stains (scale bar, 50 μm) (E) showing time-dependent heart failure, myocyte disarray, and fibrosis in iCIR^2^KO mice. (F to H) Impaired time-dependent phosphorylation of Akt in iCIR^2^KO hearts at Ser473 (#) (G) and Thr308 (# and $) (H) sites after injection of 0.001 U insulin (Ins) into the inferior vena cava as indicated (*n* = 4). Lanes in panel F were run on the same gel but were noncontiguous. GAPDH, glyceraldehyde-3-phosphate dehydrogenase; AU, arbitrary units. (I) Representative immunoblots for IRS1 and IRS2 in ventricle homogenates from mice with genotypes at time points as indicated. (J) Survival curves (*n* = 8 to 10). (K to M) Time course for ejection fraction (*n* = 6 to 13) (K), representative M-mode images (L), and representative trichrome stains (scale bar, 50 μm) (M) indicating time-dependent heart failure, myocyte disarray, and fibrosis. (N to P) Impaired time-dependent phosphorylation of Akt in iCIRS12KO hearts at Ser473 ($ and &) (O) and Thr308 ($ and &) (P) sites after injection of 0.001 U insulin into the inferior vena cava as indicated (*n* = 4). Lanes in panel N were run on the same gel but were noncontiguous. *, *P* < 0.05 versus WT at same time point or WT at same insulin concentration; †, *P* < 0.05 versus same genotype and time point with no insulin.

Cardiomyocyte-specific deletion of the IR and the IGF1R in iCIR^2^KO mice following doxycycline administration was confirmed by immunoblot analysis (Fig. 1A; see also Fig. S1A in the supplemental material). iCIR^2^KO mice exhibited increased mortality (Fig. 1B), time-dependent DCM, and cardiomyocyte disarray (Fig. 1C to E and Tables S1 to S3). Contractile function was not altered in iCIR^2^KO mice before gene deletion relative to that in wildtype (WT) controls. Basal Akt phosphorylation (Ser473 and Thr308 sites) was unchanged compared to that in WT controls 3 days and 1 week after doxycycline treatment. Insulin-stimulated Akt phosphorylation (Ser473 and Thr308 sites) was attenuated at the 1-week time point (Fig. 1F to H). Similarly, immunoblot analysis indicated cardiomyocyte-specific deletion of both IRS1 and IRS2 in iCIRS12KO mice after doxycycline treatment (Fig. 1I and Fig. S2A). Like in iCIR^2^KO mice, we observed increased mortality, time-dependent left ventricular dilation, and contractile dysfunction and myocyte disarray in iCIRS12KO mice (Fig. 1J to M and Tables S4 and S5). As observed for iCIR^2^KO mice, basal Akt phosphorylation (Ser473 and Thr308) was preserved relative to that in WT controls at 3 days and 1 week after doxycycline administration. Insulin-stimulated Akt phosphorylation was decreased by 1 week (Fig. 1N to P). Both iCIR^2^KO and iCIRS12KO mice exhibited normal glucose tolerance (Fig. S1B and C and S2B and C) and a time-dependent increase in mRNA expression of heart failure markers (Fig. S3). Together, these data indicate that combined loss of IR/IGF1R or IRS1/IRS2 signaling in the adult heart results in impaired insulin-mediated signaling, heart failure, and increased mortality.

### Increased cell death in iCIR^2^KO and iCIRS12KO hearts.

Proapoptotic signaling and atrophic signaling were increased in iCIR^2^KO and iCIRS12KO hearts. Abundance of the proapoptotic proteins BNIP3 and cleaved caspase 9 was increased at the 1-week and 4-week time points in iCIR^2^KO hearts, while expression of the antiapoptotic protein Bcl-2 was unchanged. This was supported by increased mRNA levels of genes involved in apoptotic cell death and atrophy (*Trim63* and *Fbxo32*) at the 1-week time point. Similarly, immunoblotting and reverse transcription-PCR (RT-PCR) analysis revealed increased proapoptotic and atrophic signaling in iCIRS12KO hearts as early as 3 days after doxycycline administration (Fig. S4).

### BCAA treatment decreases autophagy but does not attenuate heart failure in iCIR^2^KO and iCIRS12KO hearts.

**FIG 2:**
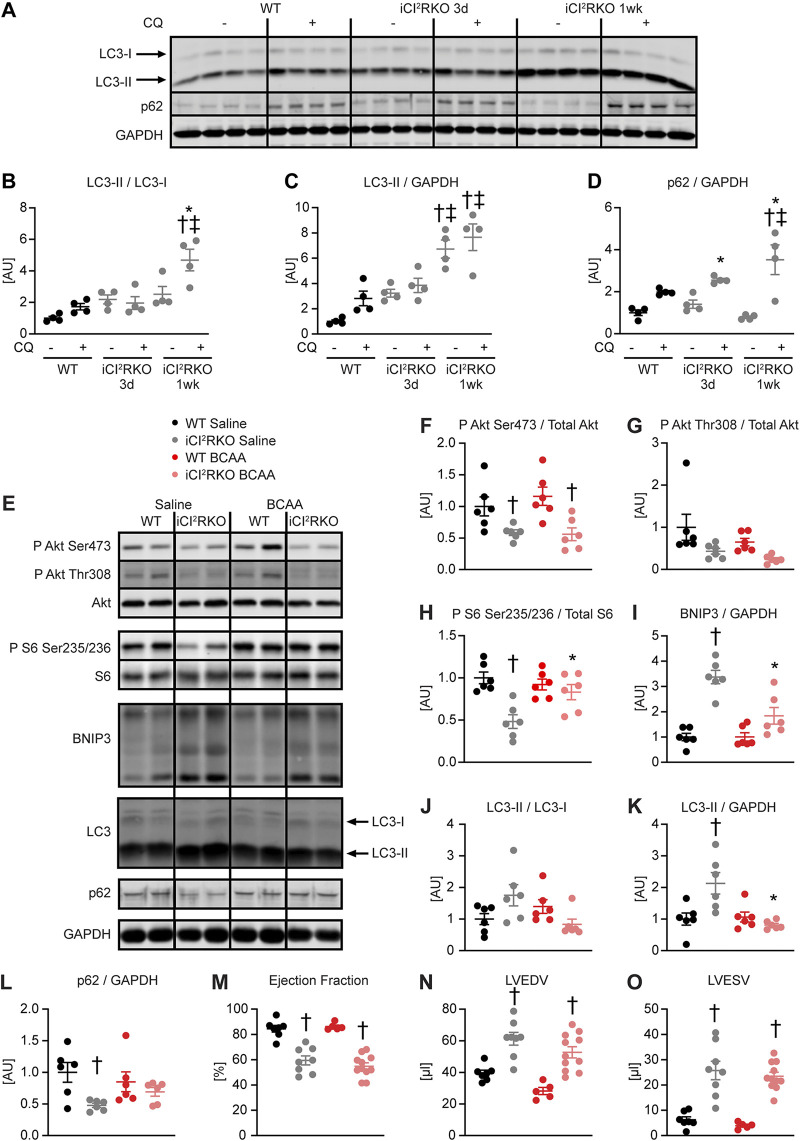
BCAA treatment decreases autophagy but does not attenuate heart failure in iCIR^2^KO mice. Two-way ANOVA was performed to analyze differences by genotype and chloroquine (CQ) injection, followed by Newman-Keuls *post hoc* analysis (#, *P* < 0.05 for CQ injection; $, *P* < 0.05 for genotype; &, *P* < 0.05 for the interaction between CQ injection and genotype; these symbol definitions apply to symbols used not in the figure itself but in the panel descriptions below). (A to D) Autophagic flux determined after CQ injection as measured by LC3-II/LC3-I (#, $, and &) (B), LC3-II/GAPDH (# and $) (C), and p62/GAPDH immunoblotting (# and &) (D). Panel A shows a representative immunoblot. Two-way ANOVA was performed to analyze differences by genotype and BCAA treatment, followed by Newman-Keuls *post hoc* analysis (¦, *P* < 0.05 for BCAA treatment; $, *P* < 0.05 for genotype; ^, *P* < 0.05 for the interaction between BCAA treatment and genotype). (E to L) Representative immunoblots (E) and quantification (F to L) from ventricle homogenates obtained from iCIR^2^KO and WT mice after saline or BCAA treatment 1 week after gene deletion. Lanes in panel E were run on the same gel but were noncontiguous. (F) P Akt Ser473/total Akt ($). (G) P Akt Thr308/total Akt ($). (H) P S6 Ser235/236/total S6 ($ and ^). (I) BNIP3/GAPDH (¦, $, and ^). (J) LC3-II/LC3-I (^). (K) LC3-II/GAPDH (¦, ^). (L) p62/GAPDH ($). *n* = 6. (M to O) Ejection fraction ($) (M), left ventricular end-diastolic volume (LVEDV; # and $) (N), and left ventricular end-systolic volume (LVESV; $) (O) determined by transthoracic echocardiography 2 weeks after gene deletion (*n* = 5 to 11). *, *P* < 0.05 versus saline, same genotype; †, *P* < 0.05 versus WT, same treatment and same time point; ‡, *P* < 0.05 versus 3-day time point, same treatment.

We previously reported that IRS signaling suppresses neonatal autophagy in the heart and that autophagy plays a critical role in the development of fatal cardiomyopathy following combined embryonic deletion of IRS1 and IRS2 (9). We therefore sought to test the hypothesis that increased autophagy contributes to heart failure in iCIR^2^KO and iCIRS12KO mice. We determined autophagic flux by LC3 immunoblotting after injection of chloroquine, which neutralizes lysosomal pH and impairs autophagosome fusion with lysosomes (16). Autophagic flux was unchanged in iCIR^2^KO hearts at the 3-day time point but was increased 1 week after doxycycline exposure (Fig. 2A to D). We previously showed that hyperactivation of mTOR signaling by BCAA suppresses autophagy *in vivo* (9). To test the hypothesis that restoration of mTOR signaling and suppression of autophagy attenuate heart failure in iCIR^2^KO hearts, iCIR^2^KO mice were intraperitoneally injected every day with a BCAA solution beginning the day of doxycycline administration.

**FIG 3:**
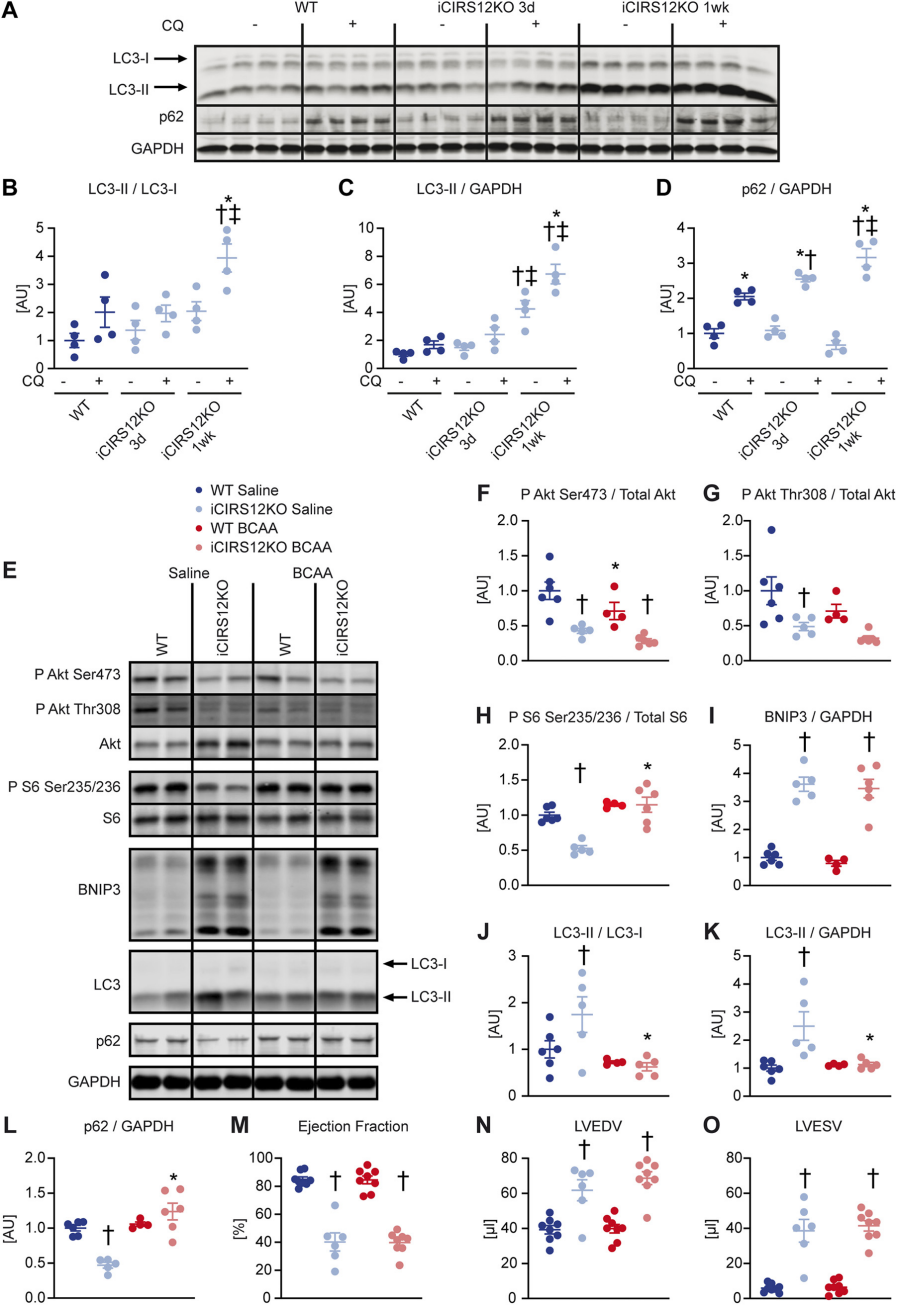
BCAA treatment decreases autophagy but does not attenuate heart failure in iCIRS12KO mice. Two-way ANOVA was performed to analyze differences by genotype and chloroquine (CQ) injection, followed by Newman-Keuls *post hoc* analysis (#, *P* < 0.05 for CQ injection; $, *P* < 0.05 for genotype; &, *P* < 0.05 for the interaction between CQ injection and genotype; these symbol definitions apply to symbols used not in the figure itself but in the panel descriptions below). (A to D) Autophagic flux determined after CQ injection as measured by LC3-II/LC3-I (# and $) (B), LC3-II/GAPDH (# and $) (C), and p62/GAPDH immunoblotting (#, $, and &) (D). Panel A shows a representative immunoblot. Two-way ANOVA was performed to analyze differences by genotype and BCAA treatment, followed by Newman-Keuls *post hoc* analysis (¦, *P* < 0.05 for BCAA treatment; $, *P* < 0.05 for genotype; ^, *P* < 0.05 for the interaction between BCAA treatment and genotype). (E to L) Representative immunoblots (E) and quantification (F to L) from ventricle homogenates obtained from iCIRS12KO and WT mice after saline or BCAA treatment 1 week after gene deletion. Lanes in panel E were run on the same gel but were noncontiguous. (F) P Akt Ser473/total Akt (¦ and $). (G) P Akt Thr308/total Akt ($). (H) P S6 Ser235/236/total S6 (¦, $, and ^). (I) BNIP3/GAPDH ($). (J) LC3-II/LC3-I (¦, $, and ^). (K) LC3-II/GAPDH (¦). (L) p62/GAPDH (¦, $, and ^). *n* = 4 to 6. (M to O) Ejection fraction (M), left ventricular end-diastolic volume (N), and left ventricular end-systolic volume (O) ($ for each) determined by transthoracic echocardiography 2 weeks after gene deletion (*n* = 6 to 8). *, *P* < 0.05 versus saline, same genotype; †, *P* < 0.05 versus WT, same treatment and same time point; ‡, *P* < 0.05 versus 3-day time point, same treatment.

To minimize the potential confounding effect of heart failure, molecular analysis was performed at the 1-week time point, when contractile function was relatively preserved (Table S6). BCAA supplementation activated mTOR signaling in iCIR^2^KO hearts to levels observed in WT controls, as evidenced by increased S6 phosphorylation. Importantly, BCAA injections suppressed autophagy in iCIR^2^KO hearts to levels observed in WT controls as assessed by LC3 immunoblotting. However, proapoptotic signaling remained increased independent of BCAA administration, as determined by BNIP3 protein levels (Fig. 2E to L). BCAA supplementation had no effect on contractile function, as determined by transthoracic echocardiography after 2 weeks of BCAA or saline treatment (Fig. 2M to O and Table S6). Similar to iCIR^2^KO mice, iCIRS12KO mice exhibited increased autophagic flux at the 1-week time point (Fig. 3A to D). BCAA administration restored mTOR signaling and decreased autophagy but had no impact on proapoptotic signaling at the 1-week time point (Fig. 3E to L). Contractile function was similarly repressed at the 2-week time point independent of BCAA supplementation (Fig. 3M to O and Table S7). Together, these data indicate that BCAA administration decreases autophagy in both iCIR^2^KO and iCIRS12KO hearts to levels observed in WT controls but does not attenuate heart failure. Thus, in contrast to the case with mice with prenatal loss of IRS1/2, mechanisms independent of increased autophagy contribute to heart failure in iCIR^2^KO and iCIRS12KO hearts.

### Contractile dysfunction precedes mitochondrial dysfunction in iCIR^2^KO and iCIRS12KO hearts.

Earlier studies by our group identified a conserved role for IR-mediated signaling in maintaining mitochondrial oxidative capacity (4, 12, 17). We therefore tested the hypothesis that loss of IR/IGF1R and IRS1/2 signaling in adult hearts would impair mitochondrial oxidative capacity to precipitate heart failure in iCIR^2^KO and iCIRS12KO hearts. The abundances of proteins involved in oxidative phosphorylation and fatty acid transport were not changed in parallel with relatively preserved activity of the mitochondrial enzymes citrate synthase (CS) and hydroxyacyl coenzyme A (CoA) dehydrogenase (HADH) in iCIR^2^KO hearts at the 3-day and 1-week time points, when contractile function was relatively unchanged (Fig. S5). Similarly, mitochondrial protein content and mitochondrial CS and HADH enzymatic activity were relatively preserved in iCIRS12KO hearts after 3 days and 1 week of doxycycline exposure (Fig. S6). CS and HADH activity significantly declined only at the 4-week time point in hearts of both genotypes at a time when overt heart failure was present. Intriguingly, protein levels of selected mitochondrial electron transport chain subunits were increased 4 weeks after doxycycline treatment. Together, these data suggest that contractile dysfunction likely precedes impairment of mitochondrial oxidative capacity in both models.

### IR/IGF1R and IRS1/2 signaling maintains SRF transcriptional activity and sarcomeric structure in the adult heart.

**FIG 4:**
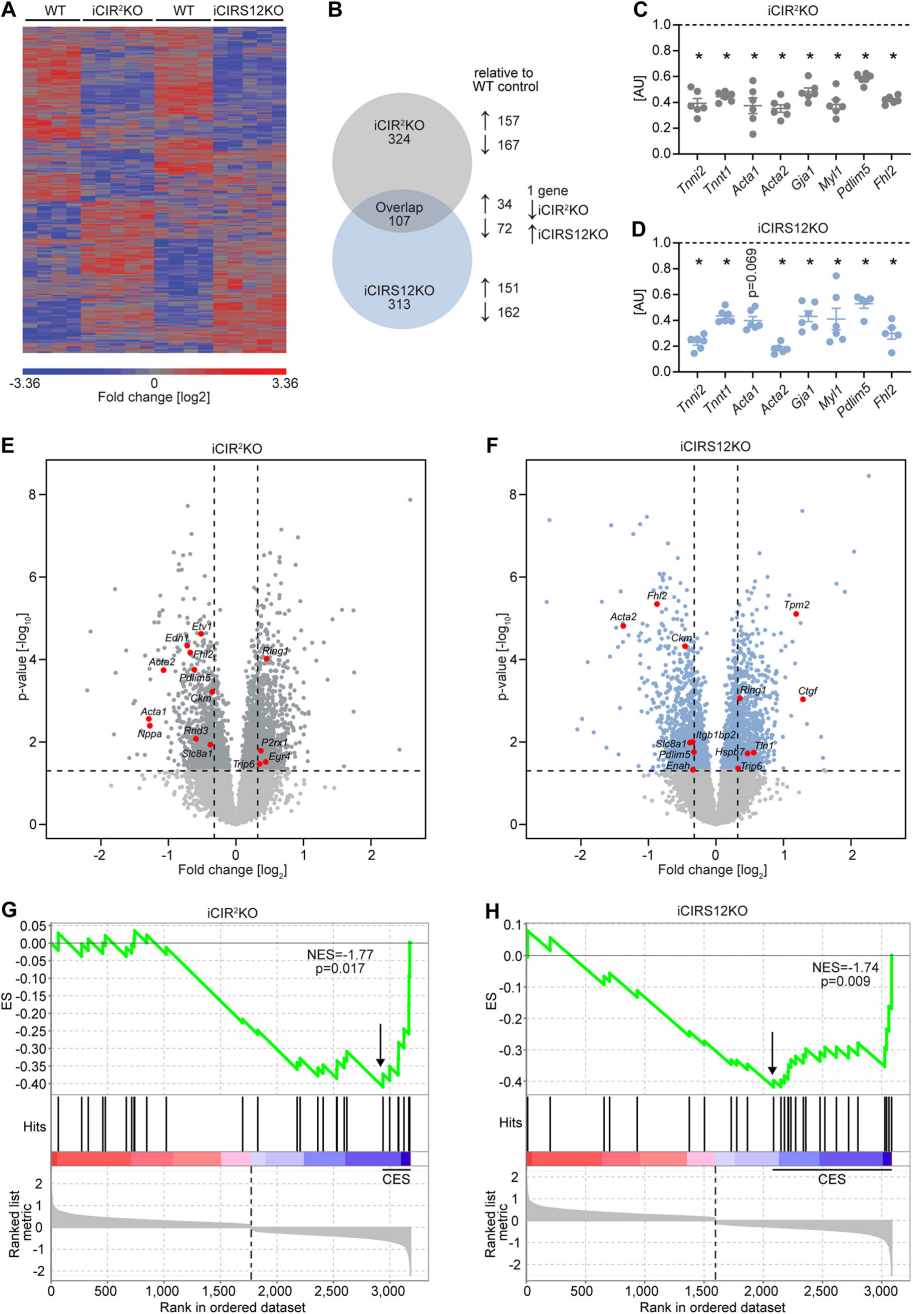
Decreased expression of genes that encode sarcomeric proteins in iCIR^2^KO and iCIRS12KO hearts 3 days post gene deletion. (A) Heat map depicting the fold change in gene expression in left ventricular apex tissue obtained from iCIR^2^KO and iCIRS12KO mice 3 days following tamoxifen-induced gene recombination relative to corresponding WT controls (|fold change| > 1.5 relative to WT controls and *P* < 0.05; *n* = 4 or 5). (B) Summary of genes that are differentially expressed relative to corresponding WT controls (|fold change| > 1.5 relative to WT controls and *P* < 0.05). (C and D) Decreased mRNA expression of genes encoding sarcomeric and gap junction proteins in iCIR^2^KO and iCIRS12KO hearts at the 3-day time point as confirmed by RT-PCR. Data are expressed as fold change relative to WT controls of the same age (assigned as 1.0; dashed line) and normalized to *Rps16* (*n* = 5 or 6). *, *P* < 0.05 versus WT, same time point. (E and F) Volcano plots highlighting expression levels of SRF targets in iCIR^2^KO and iCIR12KO hearts. Cutoffs illustrated by dashed lines indicate a *P* value of <0.05 (equals −log_10_ of ~1.30) and a |fold change| of >1.25 (equals |log_2_ fold change| of ~0.322). (G and H) Enrichment plots for SRF target genes by gene set enrichment analysis (GSEA) in iCIR^2^KO and iCIRS12KO hearts, with the *y* axis representing enrichment scores (ES) and the *x* axis indicating SRF target genes represented in the gene set (indicated as “hits”). The enrichment profile depicted by the green line connects ES and genes. NES, normalized enrichment score. The arrow indicates the point of maximal distance of the ES from the baseline as assessed by genes of the core enrichment set (CES). Lower plots in gray present all significant genes in rank order according to the signal-to-noise metric (*n* = 3,184 for iCIR^2^KO hearts and *n* = 3,085 for iCIRS12KO hearts; cutoff, *P* < 0.05). The dashed line separates SRF target genes that are positively (red) and negatively (blue) correlated with loss of cardiac IR/IGF1R signaling and IRS1/IRS2 signaling, respectively, with the colored band indicating the degree of correlation.

**FIG 5:**
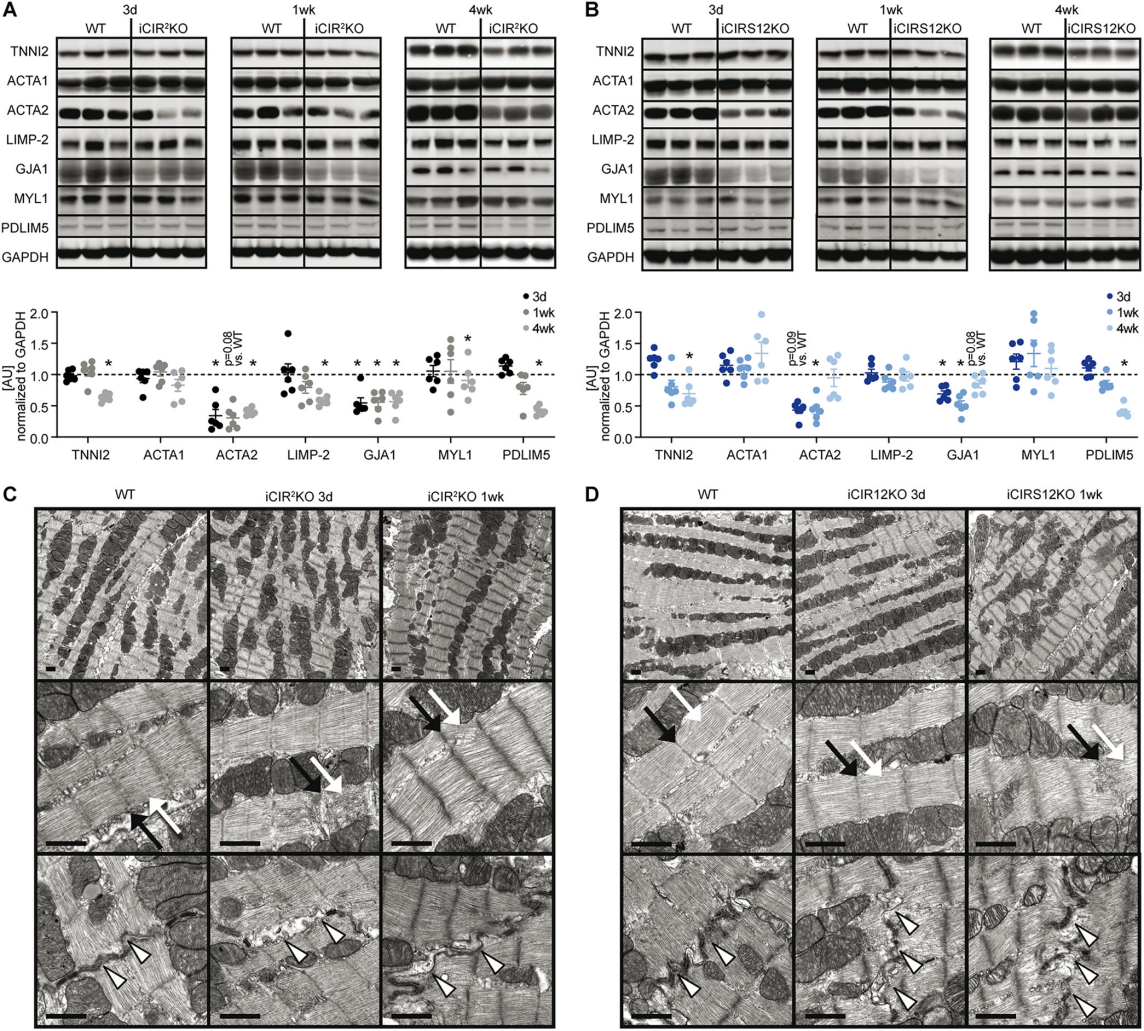
Disruption of sarcomeric integrity and cardiac structure in iCIR^2^KO and iCIRS12KO hearts. (A and B) Representative immunoblots and quantification from ventricle homogenates obtained from iCIR^2^KO and iCIRS12KO mice at time points as indicated. Data are expressed as fold change relative to WT controls at the same time point (assigned as 1.0; dashed line, *n* = 6). TNNI2, troponin I type 2; ACTA1, alpha 1 skeletal muscle actin; ACTA2, alpha 2 smooth muscle actin; LIMP-2, lysosome membrane protein 2; GJA1, gap junction protein alpha 1; MYL1, myosin light chain 1. *, *P* < 0.05 versus WT, same time point. (C and D) Representative electron micrographs of iCIR^2^KO (C) and iCIRS12KO (D) hearts 3 days and 1 week after gene deletion showing disrupted Z lines (indicated by black arrows), M lines (white arrows), and intercalated discs (arrowheads). Scale bars, 1 μm.

To identify additional mechanisms that could contribute to heart failure in iCIR^2^KO and iCIRS12KO mice, we performed microarray gene expression analysis. These experiments were performed at the 3-day time point to avoid the confounding effect of contractile dysfunction. Gene profiling analysis revealed significant differences in gene expression in iCIR^2^KO and iCIRS12KO mice (Fig. 4A and B). Of 30,854 probe sets detected on the microarray, 431 genes were differentially regulated in iCIR^2^KO hearts and 420 in iCIRS12KO hearts (*P* < 0.05 and |fold change| > 1.5 relative to values for WT controls). Gene ontology (GO) enrichment analysis identified a decrease in the abundance of transcripts associated with cardiac structure and sarcomeric integrity in iCIR^2^KO and iCIRS12KO hearts, which was confirmed by RT-PCR analysis (Fig. 4C and D). We next performed analysis with the Ingenuity Pathway Analysis (IPA) tool to identify transcriptional regulators that are responsible for this observation. Our analysis revealed impaired SRF transcriptional activity in iCIR^2^KO and iCIRS12KO hearts. SRF is a master regulator of genes that encode cardiac structural proteins (18) and is required to maintain the expression of these cardiac structure genes in the embryonic and the adult heart (19–21). Expression of SRF target genes in iCIR^2^KO and iCIRS12KO hearts is presented in Fig. 4E and F (cutoffs: |fold change| of >1.25 and *P* of <0.05) and indicates reduced expression of SRF target genes involved in sarcomeric structure in both models. Gene set enrichment analysis (GSEA) identified significant repression of SRF target genes (22) in iCIR^2^KO (normalized enrichment score [NES] of −1.77; *P* = 0.017) and iCIRS12KO (NES of −1.74; *P* = 0.009) hearts (Fig. 4G and H). Immunoblotting confirmed decreased abundance of sarcomeric proteins such as troponin isoforms, proteins associated with sarcomeric integrity such as PDLIM5, and gap junctions as early as 3 days in both models (Fig. 5A and B). Electron micrographs prepared from iCIR^2^KO and iCIRS12KO hearts revealed evidence of disrupted sarcomeric and gap junction structure as early as 3 days after doxycycline administration (Fig. 5C and D). Together, these data identify impaired SRF transcriptional activity in iCIR^2^KO and iCIRS12KO hearts, which likely contributes to loss of sarcomeric structure leading to fatal cardiomyopathy in both models investigated.

## DISCUSSION

The present study identified an essential role for IR/IGF1R-mediated signaling in maintaining sarcomeric integrity and contractile function in the adult heart. Previous studies reported accelerated heart failure following disruption of IR/IGF1R signaling in the adult heart by inducible deletion of IRS1/2 (23) or mTOR (24–26) signaling. However, these models rapidly develop contractile dysfunction and heart failure, which provided a challenge to evaluate the mechanisms proximal to the changes observed. In the present study, we utilized two independent models to disrupt IR/IGF1R signaling in the adult heart, i.e., iCIR^2^KO and iCIRS12KO mice. We investigated the potential mechanisms contributing to heart failure as early as 3 days after gene deletion, induced by doxycycline administration. Both models reveal an essential role for IR/IGF1R and IRS1/2 signaling in regulating the expression of genes encoding cardiac structural and sarcomeric proteins. Using microarray analysis, we observed decreased transcript levels of genes encoding structural and sarcomeric proteins in the heart as early as 3 days postinduction, which preceded the onset of left ventricular dysfunction (Tables S1 and S4). This was paralleled by ultrastructural evidence of disrupted sarcomeric structure evident by electron microscopy. We hypothesize that because these changes precede contractile dysfunction, they likely contribute to the subsequent development of heart failure. These observations are supported by a previous study from our group reporting disruption of gap junctions and heart failure following combined deletion of Akt1 and Akt2 in the heart (27). We observed decreased abundance of structural proteins as early as 3 days after gene deletion (Fig. 5A and B) in both models. It is important to note that the half-life of GJA1 is several hours (28), while other cardiac structure proteins, such as TNNI2, TNNI2, ACTA1, and MYL1, have a reported half-life in the range of several days (29–33). In this context, the decrease in abundance of these proteins parallels the onset of heart failure in iCIR^2^KO and iCIRS12KO mice between 1 and 2 weeks.

Our *in silico* analysis identified impaired SRF transcriptional activity in iCIR^2^KO and iCIRS12KO hearts, which provides a potential molecular mechanism accounting for the loss of sarcomere integrity and development of heart failure in both models. Cardiomyocyte-specific deletion of SRF is embryonic lethal due to perturbed cardiac maturation (19, 20). Similarly, cardiomyocyte-specific deletion of SRF in the adult heart results in loss of sarcomeric structure and heart failure (21), which is similar to the phenotype observed in iCIR^2^KO and iCIRS12KO hearts in the present study. Furthermore, our microarray analysis revealed impaired expression of myocardin (*Myocd*), which is an SRF transcriptional coactivator (34), in both models (iCIR^2^KO, −34.4% [*P* = 3.88E−05]; iCIRS12KO, −42.0% [*P* = 2.40E−06]). *In vitro* studies with canine cardiac myoblasts reported increased myocardin expression and SRF activity following insulin stimulation. It has also been suggested that insulin increases myocardin activity by posttranslational modifications (35). These studies support our *in silico* analysis that identified a critical role for IR/IGF1R signaling for SRF-mediated transcriptional activity. However, the precise mechanisms by which IR/IGF1R signaling activates SRF transcriptional activity are currently incompletely understood and require further investigation. This important issue is of great interest for future studies.

The transcriptional profiling performed in iCIR^2^KO and iCIRS12KO mice showed decreased expression of the SRF target gene *Pdlim5*, which encodes a member of the PDZ-LIM protein family. PDZ-LIM proteins are part of the Z line, which represents the border between two sarcomeres and transmits contractile force between cardiomyocytes. PDLIM5 also interacts with the cytoskeletal protein α-actinin (36), and deletion of PDLIM5 results in DCM (37). Additionally, members of the four and a half LIM domain protein (FHL) family are critical mediators of protein-protein interactions of the cytoskeleton and gene expression was also downregulated in cardiac tissue from both iCIR^2^KO and iCIRS12KO mice. Four family FHL members exist, with expression of the SRF target *Fhl2* specific to cardiac tissue. FHL2 interacts with the sarcomeric protein titin and plays a critical role in stress signaling. FHL2 has been directly linked to the development of cardiomyopathies (38–41), including familial DCM. The proposed mechanisms comprise perturbed binding of FHL2 to titin as reported for the missense Gly48Ser mutation, which disrupts the FHL2 expression pattern in cardiomyocytes (39). The impact of FHL2 on the development of DCM is further emphasized by a yeast two-hybrid screen, which revealed that the DCM-causing titin mutation Gln4053ter disrupts binding of FHL2 to titin (40). Moreover, FHL2 expression protects against superimposed stressors, such as isoproterenol infusion (42, 43). We also observed decreased mRNA and protein expression of GJA1 (connexin 43 [Cx43]) in iCIR^2^KO and iCIRS12KO mice. GJA1 gap junctions are located at the intercalated disc, which facilitates the connection between cardiomyocytes and is required for synchronous contraction. Embryonic cardiomyocyte-specific deletion of GJA1 results in premature death and is associated with electrophysiological alterations, including prolongation of the QRS complex and irregularly thickened myocardium (44). Together, these studies strongly suggest that the repression of transcripts and proteins involved in sarcomeric integrity contributes to the development of the fatal cardiomyopathy observed in iCIR^2^KO and iCIRS12KO mice. Additional studies are required to identify the precise transcriptional mechanisms by which IR/IGF1R and IRS1/2 regulate SRF-mediated transcription and the expression of genes encoding cardiac structure proteins.

Earlier studies have described heart failure in mouse models following embryonic or perinatal deletion of essential proteins involved in the IR/IGF1R signaling pathway, i.e., IR/IGF1R (8), IRS1/2 (13–15), and mTOR (45). The proposed mechanisms include mitochondrial dysfunction, excessive autophagy, and repression of genes encoding the mitochondrial electron transport chain and fatty acid oxidation. One previous study utilized transgenic mice with combined perinatal knockout of IR and IGF1R, which was facilitated by Cre recombinase under the control of the muscle creatine kinase (MCK) promoter. This model showed altered expression of genes involved in the contractile apparatus (8), which has been suggested as a potential mechanism for the onset of heart failure. While these mice die by about 4 weeks of age, transcriptional profiling was performed at 8 and 20 days of age, when hearts were already reduced in size and showed increased expression of the fetal β-myosin heavy chain (βMHC; encoded by *Myh7*). Importantly, expression of genes encoding the contractile apparatus was increased at 20 days of age in the presence of perturbed sarcomeric structure. In the present study, *Myh7* expression was increased only in the presence of contractile dysfunction (Fig. S3 and Tables S1 and S4), which is consistent with a previous study reporting increased βMHC expression in failing hearts (46). Therefore, the altered expression of genes encoding cardiac structural proteins observed following perinatal IR/IGF1R expression could be a consequence of cardiac dysfunction. These data underscore distinct consequences of IR/IGF1R signaling on gene expression in the perinatal period relative to those in the adult heart.

Perturbed sarcomeric structure observed in iCIR^2^KO and iCIRS12KO hearts is likely a consequence of impaired IR/IGF1R/Akt signaling and not a consequence of heart failure *per se*. Moreover, the direction of insulin signaling is opposite that observed in models of pressure overload-induced cardiac hypertrophy and failure, which are characterized by increased IR/Akt-mediated signaling (47). We examined a model of decompensated left ventricular hypertrophy following transverse aortic constriction (TAC; 4-week duration, 30-gauge) with an approximate 40% reduction in fractional shortening and 65% increase in heart weight/tibia length ratio (48). As previously reported for this model (47, 49), these hearts exhibited increased Akt signaling. Importantly, expression of cardiac structure proteins was preserved post-TAC (Fig. S7). Thus, the mechanism by which loss of IR/IGF1R signaling perturbs the expression of cardiac structural proteins to induce heart failure is not a generalized mechanism that is applicable to models of heart failure induced by hemodynamic stress.

We previously reported that increased autophagy contributes to accelerated heart failure in mice with embryonic deletion of IRS1/2 (13). In the present study, we observed increased autophagic flux in both models at the 1-week time point (Fig. 2A to D and 3A to D) when contractile function was relatively preserved (Tables S6 and S7). BCAA treatment decreased autophagy in iCIR^2^KO and iCIRS12KO hearts to levels observed in WT controls; however, BCAA treatment did not attenuate the onset of heart failure (Fig. 2E to O and 3E to O and Tables S6 and S7). These data suggest that mechanisms independent of increased autophagy contribute to heart failure in iCIR^2^KO and iCIRS12KO mice. Importantly, abundances of proteins involved in cardiac structure were not influenced by BCAA supplementation (Fig. S8). Thus, distinct mechanisms mediated by IR/IGF1R and IRS1/2 contribute to heart failure following embryonic deletion versus inducible deletion of these signaling proteins in the adult heart.

We previously reported an important role for IR-mediated signaling for maintaining mitochondrial capacity (4, 12, 17). These observations were made in mice with perinatal loss of insulin signaling. This contrasts with our observations in iCIR^2^KO and iCIRS12KO mice, which exhibited preserved mitochondrial capacity despite impaired signal transduction at a time when myocardial contractility was relatively preserved, at the 3-day and 1-week time points (Fig. S5 and S6). Impaired mitochondrial oxidative capacity was observed only at the 4-week time point, which could be secondary to advanced heart failure. These data identify developmental differences in the regulation of mitochondrial oxidative capacity by IR/IGF1R and IRS1/2 signaling in the neonatal versus the adult heart.

In addition to IR and IGF1R, IRS proteins can be activated by integrins and a subset of cytokine receptors following their activation by interleukins (50). Loss of signaling through these interaction partners could potentially result in heart failure in IRS1/2-deficient hearts by mechanisms that are independent of IR/IGF1R signaling. For example, interleukin 6 (IL-6) has prosurvival effects independent of IR/IGF1R signaling and recruits IRS-1 to the IL-6 receptor complex as observed in skeletal muscle cells (51). IL-6 has been reported to activate the transcription factor signal transducer and activator of transcription 3 (STAT3). STAT3 regulates the expression of cytoprotective and antiapoptotic genes (those encoding manganese superoxide dismutase [MnSOD], Bcl-xL, and Hsp70) and hypertrophy markers (βMHC) in cardiomyocytes (52). Therefore, IRS1-transduced activation of IL-6/STAT3 signaling could also contribute to IRS-mediated prosurvival effects that are independent of IR/IGF1R signaling. We performed a detailed analysis of previously identified prosurvival signaling pathways in both iCIR^2^KO and iCIRS12KO hearts and found no differences in prosurvival signaling between the genotypes (Fig. S9). Furthermore, microarray gene expression analysis followed by a comparative GO enrichment analysis did not identify any differences in a prosurvival transcriptional signature between iCIR^2^KO and iCIRS12KO mice.

Limitations of the present study include the fact that only male iCIRS12KO mice were investigated. We observe similar phenotypes for male and female iCIR^2^KO mice (Table S2). A recent study reported that estrogen attenuates heart failure following deletion of IRS1 and IRS2 (53). In that study, embryonic cardiomyocyte-specific deletion of IRS1 and IRS2 resulted in death by the age of 6 to 8 weeks, while female mice with the same genotype survive greater than 1 year. Moreover, estrogen trended to increase survival in male mice with inducible cardiomyocyte-specific deletion of IRS1 and IRS2 based on the α-myosin heavy chain (αMHC)-MerCreMer (MCM) system. These data suggest that estrogen attenuates heart failure in the presence of perturbed cardiac insulin signaling and provides a mechanism for the gender difference in the prevalence of cardiovascular disease and type 2 diabetes. This contrasts to our studies in iCIR^2^KO mice and mice with embryonic cardiomyocyte-specific deletion of IRS1 or IRS2 and deletion of both IRS isoforms, which show similar phenotypes for male and female mice (13; C. Riehle and E. D. Abel, unpublished observations). Moreover, the presence of overlapping mechanisms resulting in heart failure in iCIR^2^KO and iCIRS12KO mice renders it likely that our female iCIRS12KO mice will also reveal a heart failure phenotype. The difference between these independent studies might emanate from differences in the genetic backgrounds of the animals investigated and the composition of the mouse chow used. Another limitation is that gene profiling by microarray analysis was performed in left ventricular tissue, which comprises different cell types, including cardiomyocytes, fibroblasts, and endothelial cells. Thus, our studies cannot discern the impact of cardiomyocyte-specific deletion of IRS1/IRS2 or IR/IGF1R on gene expression of distinct cellular populations of the heart. Recent advances in RNA sequencing technologies allow investigation of this important aspect at single-cell resolution, which is of interest for the design of future studies.

**FIG 6:**
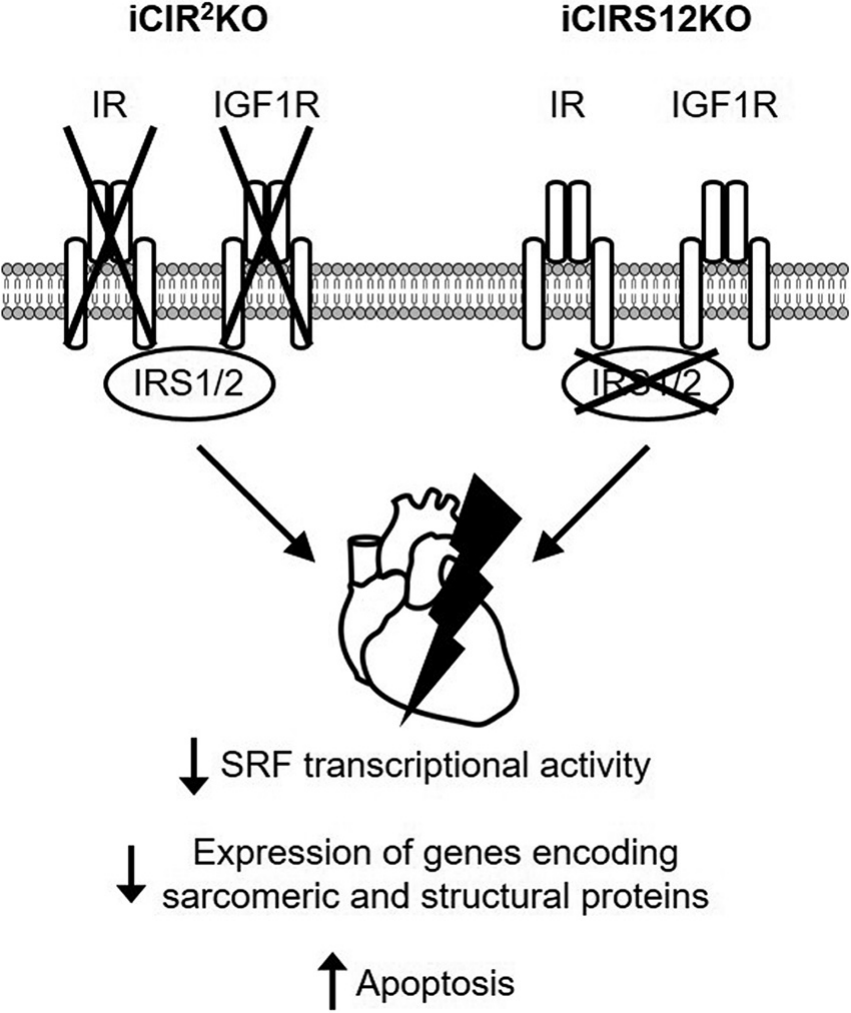
Mechanisms for the rapid onset of heart failure in iCIR^2^KO and iCIRS12KO mice. Disruption of insulin and IGF1 signaling in iCIR^2^KO and iCIRS12KO hearts attenuates SRF transcriptional activity, which results in impaired transcription of genes encoding sarcomeric and structural proteins. This mechanism in concert with increased apoptosis leads to the rapid onset of heart failure.

In summary, these data show that signals transduced by IR/IGF1R and IRS1/2 are essential for maintaining cardiac structure and contractile function in the adult heart. Pathomechanisms contributing to accelerated heart failure in iCIR^2^KO and iCIRS12KO mice are independent of increased autophagy and mitochondrial dysfunction. Thus, distinct mechanisms contribute to heart failure following disruption of IR/IGF1R signaling in the embryonic relative to the adult heart (Fig. 6). Moreover, we identify an essential role for an SRF-mediated transcriptional program by which IR/IGF1R signaling coordinately regulates the expression of genes that encode proteins that maintain sarcomeric integrity and cardiac structure in the adult heart.

## MATERIALS AND METHODS

### Animals.

Mice with inducible cardiomyocyte-specific deletion of IRS1/IRS2 (iCIRS12KO) or IR/IGF1R-DKO (iCI^2^RKO) were obtained by generating compound transgenic mice harboring a *tetO-Cre* transgene (strain no. 6234; Jackson Laboratories), a reverse tetracycline transactivator under the control of the αMHC promoter (αMHC-rtTA) (54), and alleles flanked by *loxP* sites for *Irs1* and *Irs2* or for *Ir* and *Igf1r*. iCIRS12KO mice had the genotype *Irs1^lox/lox^ Irs2^lox/lox^ tetO-Cre*^+/−^ *αMHC-rtTA*^+/−^; iCI^2^RKO mice had the genotype *Ir^lox/lox^ Igf1r^lox/lox^ tetO-Cre*^+/−^ *αMHC-rtTA*^+/−^. The generation of transgenic *Irs1^lox/lox^*, *Irs2^lox/lox^*, *Ir^lox/lox^*, and *Igf1r^lox/lox^* mice has been described previously (16, 55–57). *Irs1^lox/lox^ Irs2^lox/lox^* mice were used as WT controls for iCIRS12KO, and *Ir^lox/lox^ Igfir^lox/lox^* mice were used as WT controls for iCI^2^RKO. All mice were injected intraperitoneally at 8 weeks of age with 0.1 mg doxycycline hyclate (Sigma-Aldrich, St. Louis, MO) and then placed on doxycycline chow (1 g/kg body weight; Bio-serv, Frenchtown, NJ). After 1 week, mice were switched to regular rodent chow. All mice were on a mixed genetic background, and littermate controls were used. Animals were housed with a 12-h light/12-h dark cycle at 22°C with free access to food and water. All experiments were performed in accordance with protocols approved by the Institutional Animal Care and Use Committee of the Carver College of Medicine of the University of Iowa. Data presented are from male mice unless otherwise indicated.

### Quantitative RT-PCR.

Total RNA was extracted from hearts with TRIzol reagent (Invitrogen Corporation, Carlsbad, CA) and reverse transcribed. Quantitative real-time PCRs were performed using SYBR green I and ROX internal reference as previously described (13). Primer sequences are listed in the supplemental material.

### BCAA supplementation.

Branched-chain amino acids (BCAA) solution was supplemented daily via intraperitoneal injections starting at the same time as doxycycline initiation at a dosage of 2 mL/day. The solution contained the following concentrations of amino acids (in grams per liter): isoleucine, 6.0; leucine, 12.0; valine, 7.2; and arginine, 6.04. The pH was 7.4. All amino acids were purchased from Sigma-Aldrich (St. Louis, MO). Saline was used as a control.

### Transthoracic echocardiography.

Transthoracic echocardiography was performed on nonanesthetized mice. Parasternal short- and long-axis B-mode images were obtained using high-frequency echocardiography (Vevo 2100; VisualSonics, Toronto, Canada) to determine left ventricular volumes and dimensions (58). Echocardiographic images were analyzed by an operator blinded to mouse genotype. Endocardial and epicardial borders were determined in short-axis plane at end-diastole and end-systole. Left ventricular end-diastolic and end-systolic volumes and ejection fraction were determined using the biplane area-length method (59). For illustrative purposes (Fig. 1D and L), M-mode images were generated *post hoc* from long-axis B-mode images, with the line of interrogation placed transverse to the long axis at the level of papillary muscle tips, using vendor software designed for that purpose.

### Immunoblotting.

For immunoblotting analysis, total protein was extracted from left ventricular tissue homogenized in lysis buffer (50 mmol/L HEPES, 150 mmol/L NaCl, 10% glycerol, 1% Triton X-100, 1.5 mmol/L MgCl_2_, 1 mmol/L EGTA, 10 mmol/L sodium pyrophosphate, 100 mmol/L sodium fluoride, 100 μmol/L sodium vanadate, 1 mmol/L phenylmethylsulfonyl fluoride [PMSF], 10 μg/mL aprotinin, and 10 μg/mL leupeptin) using a tissue homogenizer (TissueLyser II; Qiagen, Valencia, CA) (12). Proteins were resolved by SDS-PAGE and electrotransferred to nitrocellulose (IRS1 and IRS2) or polyvinylidene difluoride (PVDF) membranes (other targets). Protein detection was carried out with Alexa Fluor secondary antibodies (Invitrogen Corporation, Carlsbad, CA), and fluorescence was quantified using a LI-COR Odyssey imager (Lincoln, NE). Primary antibodies used are listed in the supplemental material.

### Assessment of autophagic flux.

Mice were injected with chloroquine (Sigma-Aldrich, St. Louis, MO) at a dose of 30 mg/kg body weight 48 h prior to harvest and 30 mg/kg body weight 24 h prior to harvest. Mice were sacrificed after a 6-h fast starting at 7 a.m. and 2 h after the final injection of chloroquine (40 mg/kg body weight). Heart tissues were then snap-frozen and subjected to immunoblot analysis (13).

### Evaluation of insulin-stimulated signal transduction.

Mice were anesthetized with isoflurane after a 6-h fast starting at 7 a.m. and were injected with 0.001 U insulin or saline via the inferior vena cava. Hearts were harvested 5 min post-injection, snap-frozen in liquid nitrogen, and processed for immunoblot analysis.

### Histology.

Myocardial fragments were embedded in paraffin, portioned into 5-μm-thick sections, and stained with trichrome as previously described (60).

### Electron microscopy.

Left ventricular tissue was incubated in fixation buffer containing 2.5% glutaraldehyde and 0.1 M sodium cacodylate-paraformaldehyde for at least 1 day. Samples were washed three times for 20 min in 0.1 M sodium cacodylate (pH 7.2), fixed in 4% osmium tetroxide, and washed in 0.1 M sodium cacodylate, followed by water and 2.5% uranyl acetate. Next, samples were dehydrated using a graded series of ethanol washes (50% up to 100%), embedded in Spurr’s plastic, and processed for electron microscopy. Following ultramicrotomy, sections were examined using a JEOL JEM-1230 transmission electron microscope (JEOL, Peabody, MA). Digital images were collected with an UltraScan 1000 2k × 2k charge-coupled-device (CCD) camera (Gatan, Pleasanton, CA) (61).

### Microarray analysis.

Microarray analysis was performed on RNA extracted from left ventricular apex tissue following 3 days of doxycycline exposure. Gene expression was determined using MouseWG-6 v2.0 Expression BeadChips (Illumina, San Diego, CA) and performed by the Iowa Institute of Human Genetics (IIHG) Genomics Division. Differential gene expression and statistical analyses (analysis of variance [ANOVA]) were performed using Partek Genomics Suite (v6.6) and the gene expression workflow. *P* values were adjusted using the Benjamini-Hochberg procedure. Differentially expressed genes in the knockout groups were defined by a *P* value of <0.05 and |fold change| of >1.5 relative to the values for the corresponding WT controls. Functional analysis was performed using the Ingenuity Pathway Analysis (IPA) tool (Qiagen Inc. [https://www.qiagenbioinformatics.com/products/ingenuity-pathway-analysis/]). SRF targets were classified based on a previously published data set (22). Volcano plots were generated with the statistical programming language R (R Core Team). Gene set enrichment analysis (GSEA) was performed with software version 4.1.0 (62).

### Glucose tolerance tests and insulin tolerance tests.

For glucose tolerance tests, mice were fasted for 6 h starting at 6 a.m. and were injected intraperitoneally with 1 g glucose/kg body weight. Insulin tolerance tests were performed on random fed animals by intraperitoneal injection of 0.75 U insulin/kg body weight. Blood glucose concentrations were measured using a glucometer (Glucometer Elite; Bayer, Tarrytown, NY). Area under the curve (AUC) was calculated using GraphPad Prism software (GraphPad, San Diego, CA).

### Statistical analysis.

Data are expressed as means ± standard errors of the means (SEM). Unpaired Student’s *t* tests were used to determine *P* values when comparing two groups. For multigroup comparisons, differences were analyzed by two-way ANOVA, and significance was assessed by Newman-Keuls *post hoc* analysis. Statistical calculations were performed using GraphPad Prism software (GraphPad, San Diego, CA). A *P* value of <0.05 was considered significantly different.
