# Supplementary material for: Insulin and Insulin-Like Growth Factor 1 Signaling Preserves Sarcomere Integrity in the Adult Heart
Source: Mol Cell Biol. 2022 Sep 20;42(10):e00163-22. doi: 10.1128/mcb.00163-22 (PMC9583714; doi:10.1128/mcb.00163-22)
Supplement: Supplemental file 1 — Supplemental material. Download mcb.00163-22-s0001.pdf, PDF file, 2.7 MB [file mcb.00163-22-s0001.pdf]

SUPPLEMENTAL MATERIAL

for

Insulin and IGF1 Signaling Preserves Sarcomere Integrity in the Adult Heart

Christian Riehle, MD[1,2,7], Eric T. Weatherford, PhD[1,7], Nicholas S. McCarty, MS[1], Alec Seei, DO[1], Bharat P. Jaishy, PhD[1], Rajkumar Manivel, MD[1], Paolo Galuppo, PhD[2], Chantal Allamargot, PhD[3], Tariq Hameed, MD[4], Ryan L. Boudreau, PhD[4,5], Johann Bauersachs, MD[2], Robert M. Weiss, MD[4,5], E. Dale Abel, MBBS, DPhil[1,5,6]

[1] Fraternal Order of Eagles Diabetes Research Center and Division of Endocrinology and Metabolism, Carver College of Medicine, University of Iowa, Iowa City, IA 52242, USA
[2] Department of Cardiology and Angiology, Hannover Medical School, Hannover, Germany
[3] Central Microscopy Research Facility, Carver College of Medicine, University of Iowa, Iowa City, IA 52242, USA
[4] Division of Cardiovascular Medicine, Carver College of Medicine, University of Iowa, Iowa City, IA 52242, USA
[5] Department of Internal Medicine, Carver College of Medicine, University of Iowa, Iowa City, IA 52242, USA
[6] Current Address, Department of Medicine, David Geffen School of Medicine, UCLA, CA, USA
[7] Authors contributed equally.

Supplemental Methods

Primers used for quantitative RT-PCR

Gene Name<br>Gene Sequence of forward and reverse primers (5'→ 3')<br>GenBank Accession Number
Actin, alpha 1, skeletal muscle *(Acta1)*<br>CCTGTATGCCAACAACGTCA<br>CTCGTCGTACTCCTGCTTGG<br>NM_001272041.1
Actin, alpha 2, smooth muscle, aorta *(Acta2)*<br>GTCCCAGACATCAGGGAGTAA<br>TCGGATACTTCAGCGTCAGGA<br>NM_007392.3
BCL2/adenovirus E1B interacting protein 3 *(Bnip3)*<br>TTGGCGAGAAAAACAGCAC<br>GCTGAGAAAATTCCCCCTTT<br>NM_009760.4
BCL2-like 1 */ Bcl-XL (Bcl2l1)*<br>GACAAGGAGATGCAGGTATTGG<br>TCCCGTAGAGATCCACAAAAGT<br>NM_001289716.1
F-box protein 32 / Atrogin-1 / MAFbx *(Fbxo32)*<br>GCTGGATTGGAAGAAGATGTATT<br>TTGAGGGGAAAGTGAGACG<br>NM_026346.3
Four and a half LIM domains 2 *(Fhl2)*<br>TTAAAGCAACCCAATGAAGCCC<br>GCTGACACCTGTCTTTCAGCA<br>NM_001289533.1
Gap junction protein, alpha 1 *(Gja1)*<br>ACAGCGGTTGAGTCAGCTTG<br>GAGAGATGGGGAAGGACTTGT<br>NM_010288.3
Growth hormone receptor *(Ghr)*<br>ACAGTGCCTACTTTTGTGAGTC<br>GTAGTGGTAAGGCTTTCTGTGG<br>NM_010284.3
Interleukin 6 receptor *(Il6r)*<br>CCTGAGACTCAAGCAGAAATGG<br>AGAAGGAAGGTCGGCTTCAGT<br>NM_010559.2
Myosin, heavy polypeptide 7, cardiac muscle, beta / βMHC *(Myh7)*<br>CATTCTCCTGCTGTTTCCTTAC<br>CATGGCTGAGCCTTGGAT<br>NM_080728.2
Myosin, light polypeptide 1 *(Myl1)*<br>AGGAGCAACAGGAGGACTTCA<br>CTCTGCATTGGTGGGATTGGT<br>NM_021285.3

Natriuretic peptide precursor type A *(Nppa)*<br>ATGGGCTCCTTCTCCATCA<br>CCTGCTTCCTCAGTCTGCTC<br>NM_008725.2
Natriuretic peptide precursor type B *(Nppb)*<br>GGATCTCCTGAAGGTGCTGT<br>TTCTTTTGTGAGGCCTTGGT<br>NM_008726.5
PDZ and LIM domain 5 *(Pdlim5)*<br>TAAGGCTTGTACGGGCTCCT<br>GGATCCTCTTCTCTGTCCCTCT<br>NM_022554.3
Peroxisome proliferative activated receptor, gamma, coactivator 1 alpha *(Ppargc1a)*<br>GTAAATCTGCGGGATGATGG<br>AGCAGGGTCAAAATCGTCTG<br>NM_008904.2
Ribosomal protein S16 *(Rps16)*<br>TGCTGGTGTGGATATTCGGG<br>CCTTGAGATGGGCTTATCGG<br>NM_013647.2
Superoxide dismutase 2, mitochondrial *(Sod2)*<br>ACAACTCAGGTCGCTCTTCA<br>GAACCTTGGACTCCCACAGA<br>NM_013671.3
Tripartite motif-containing 63 / MuRF1 *(Trim63)*<br>GTGTGAGGTGCCTACTTGCTC<br>GCTCAGTCTTCTGTCCTTGGA<br>NM_001039048.2
Troponin I, skeletal, fast 2 *(Tnni2)*<br>TTCGGAGGGTGCGTATGTCT<br>GTCCCGTTCCTTCTCAGTGT<br>NM_009405.2
Troponin T1, skeletal, slow *(Tnnt1)*<br>TGTGCTCTACAACCGCATCA<br>AGTTACAGATGGGACACGCT<br>NM_001277903.1

Primary antibodies used for immunoblotting

Antigen	Company
α-Tubulin	Sigma-Aldrich, St. Louis, MO
ACTA1	Sigma-Aldrich, St. Louis, MO
ACTA2	Abcam, Cambridge, MA
ATP5A	Abcam, Cambridge, MA
Bcl-2	Cell Signaling, Danvers, MA
BNIP3	Cell Signaling, Danvers, MA
Cleaved Caspase 9	Cell Signaling, Danvers, MA
GAPDH	Cell Signaling, Danvers, MA
GJA1	Sigma-Aldrich, St. Louis, MO
HSP70	Enzo Life Sciences, Farmingdale, NY
IGF1R	Cell Signaling, Danvers, MA
IR	Cell Signaling, Danvers, MA
IRS1	Millipore, Billerica, MA
IRS2	Cell Signaling, Danvers, MA
LC3	Sigma-Aldrich, St. Louis, MO
LIMP-2	Abcam, Cambridge, MA
MnSOD	Enzo Life Sciences, Farmingdale, NY
MYL1	Sigma-Aldrich, St. Louis, MO
NDUFA9	Abcam, Cambridge, MA
P Akt Ser473	Cell Signaling, Danvers, MA
P Akt Thr308	Cell Signaling, Danvers, MA
P S6 Ser235/236	Cell Signaling, Danvers, MA
P STAT3 Tyr705	Cell Signaling, Danvers, MA
PDLIM5	Abcam, Cambridge, MA
p62	Santa Cruz Biotechnology, Santa Cruz, CA
SDHA	Abcam, Cambridge, MA
TNNI2	Cell Signaling, Danvers, MA
TNNT1	Cell Signaling, Danvers, MA
Total Akt	Cell Signaling, Danvers, MA
Total S6	Cell Signaling, Danvers, MA
Total STAT3	Cell Signaling, Danvers, MA
UQCRC1	Abcam, Cambridge, MA
VDAC	Thermo Scientific, Waltham, MA

Supplemental Figures

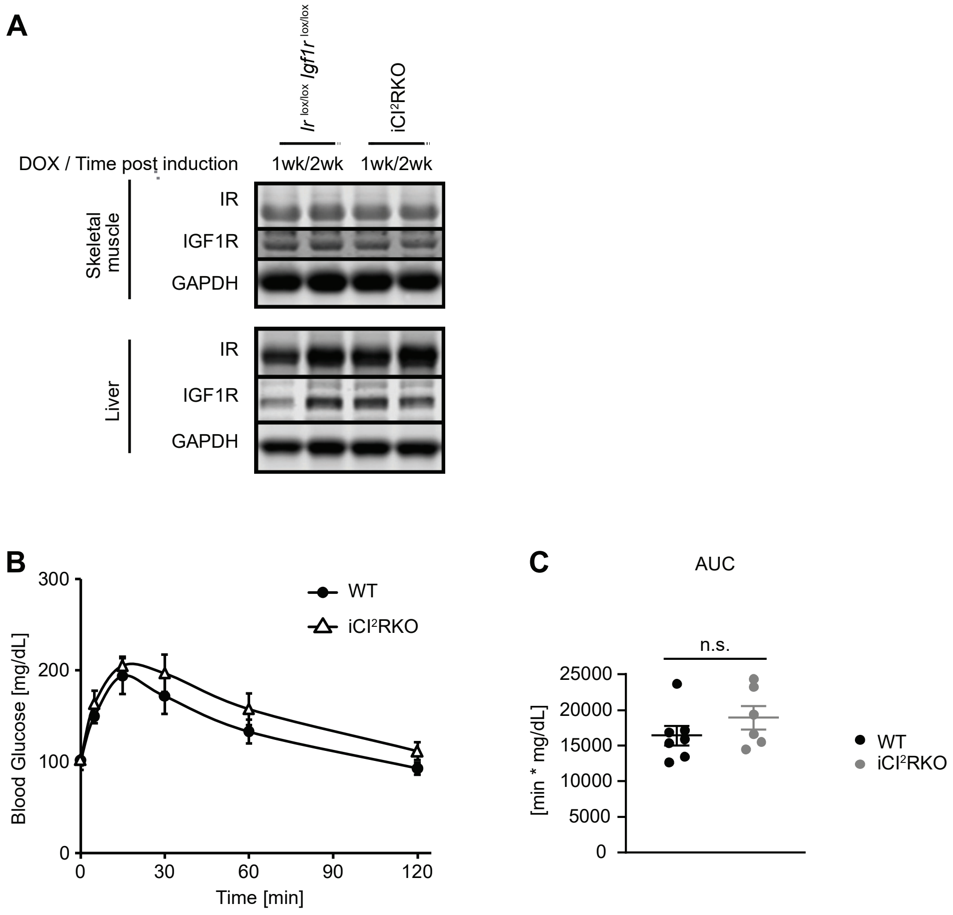

Supplemental Figure 1. *IR/IGF1R protein levels in skeletal muscle and liver and glucose tolerance tests in iCIR²KO mice.*
(A) IR and IGF1R protein levels in tissues obtained from iCIR²KO mice as indicated. (B/C) Glucose tolerance tests at 2 weeks post gene deletion. Blood glucose was measured at the indicated time points post injection. AUC, area under the curve; n.s., no significant difference observed. Data are reported as mean values ± SEM, n=6-7.

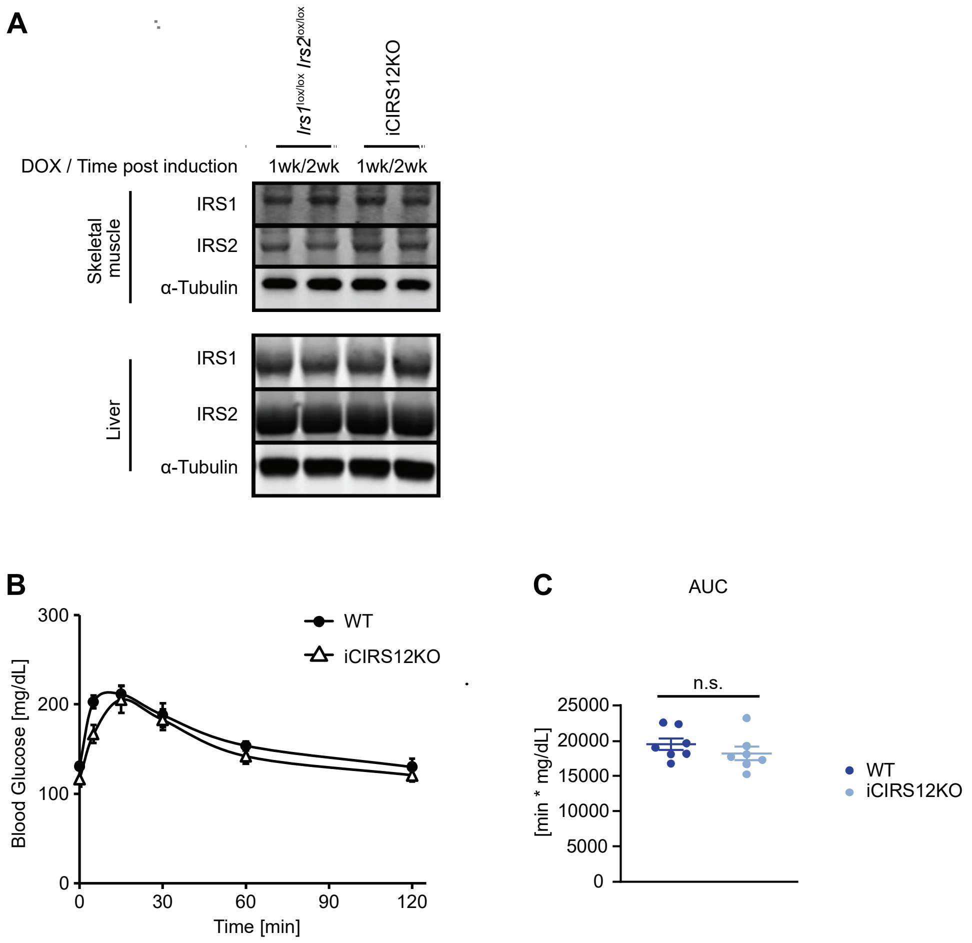

Supplemental Figure 2. *IRS protein levels in skeletal muscle and liver and glucose tolerance tests in iCIRS12KO mice.*
(A) IRS1 and IRS2 protein levels in tissues obtained from iCIRS12KO mice as indicated. (B/C) Glucose tolerance tests at 2 weeks post gene deletion. Blood glucose was measured at the indicated time points post injection. AUC, area under the curve; n.s., no significant difference observed. Data are reported as mean values ± SEM, n=7.

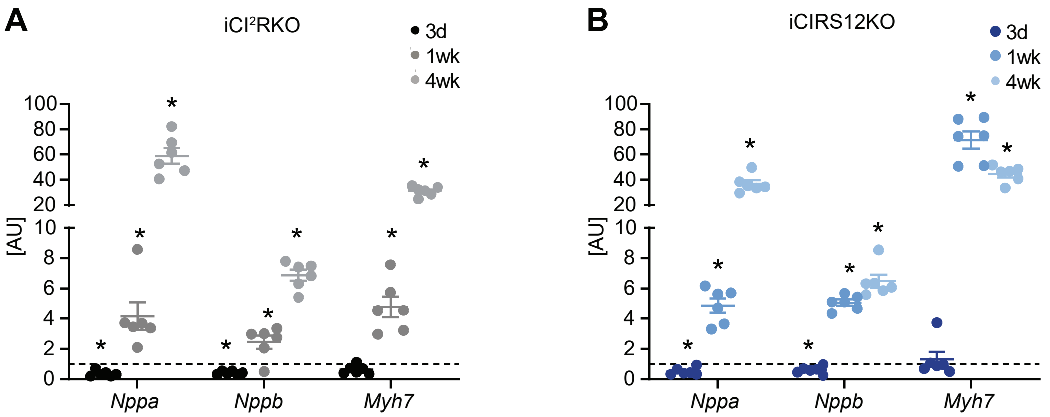

Supplemental Figure 3. *Time-dependent increase in mRNA expression of heart failure markers in iCIR²KO and iCIRS12KO hearts.*

mRNA expression in (A) iCIR²KO and (B) iCIRS12KO hearts at time points as indicated. Data are presented as mean values ± SEM and as fold change relative to WT controls at the same time point (assigned as 1.0; dashed line) and normalized to *Rps16*. * $p<0.05$ vs. WT same time point.

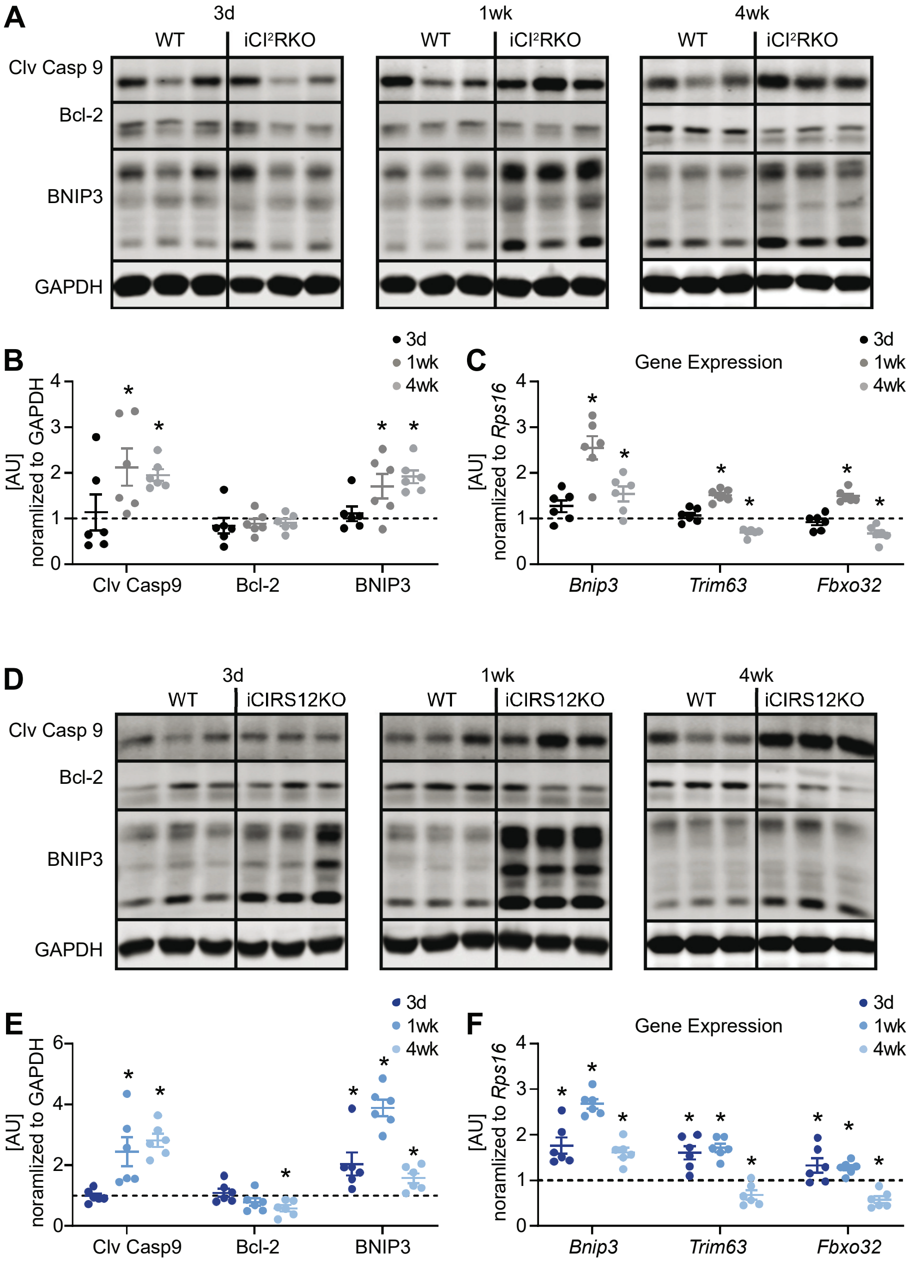

Supplemental Figure 4. *Increased cell death and atrophic signaling in iCIR²KO and iCIRS12KO hearts.*

(A) Representative immunoblots (B) and densitometric quantification for proteins involved in cell death in iCIR²KO hearts at time points as indicated. (C) mRNA expression of genes involved in cell death and atrophy normalized to *Rps16* (n=6). (D) Representative immunoblots (E) and densitometric quantification in iCIRS12KO hearts at time points as indicated. (F) mRNA expression in iCIRS12KO hearts normalized to *Rps16* (n=6). Data are expressed as fold change relative to WT controls at the same time point (assigned as 1.0; dashed line). * p<0.05 vs. WT same time point (n=6 each).

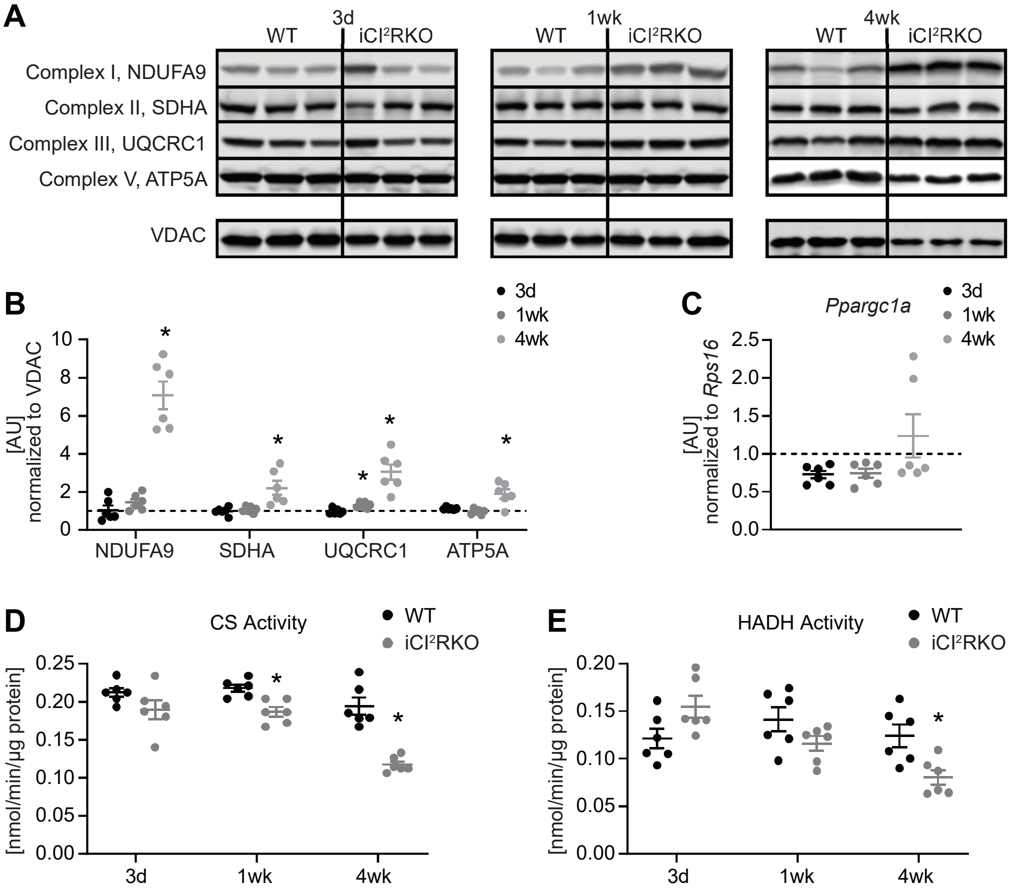

Supplemental Figure 5. *Contractile dysfunction precedes mitochondrial dysfunction in iCIR^{2}KO hearts.*

(A) Representative immunoblots and (B) densitometric analysis of electron transport chain subunits NDUFA9 (complex I), SDHA (complex II), UQCRC1 (complex III), and ATP5A (complex V) normalized to VDAC. Data are presented as fold change relative to WT controls at the same time point (assigned as 1.0; dashed line). (C) mRNA expression of the transcriptional coactivator PGC-1α *(Ppargc1a)* normalized to *Rps16* presented as fold change relative to WT controls at the same time point (assigned as 1.0; dashed line). (D) Citrate synthase (CS) and (E) hydroxyacyl-CoA dehydrogenase (HADH) enzymatic activity. Data are reported as mean values ± SEM, n=6. * $p<0.05$ vs. WT same time point.

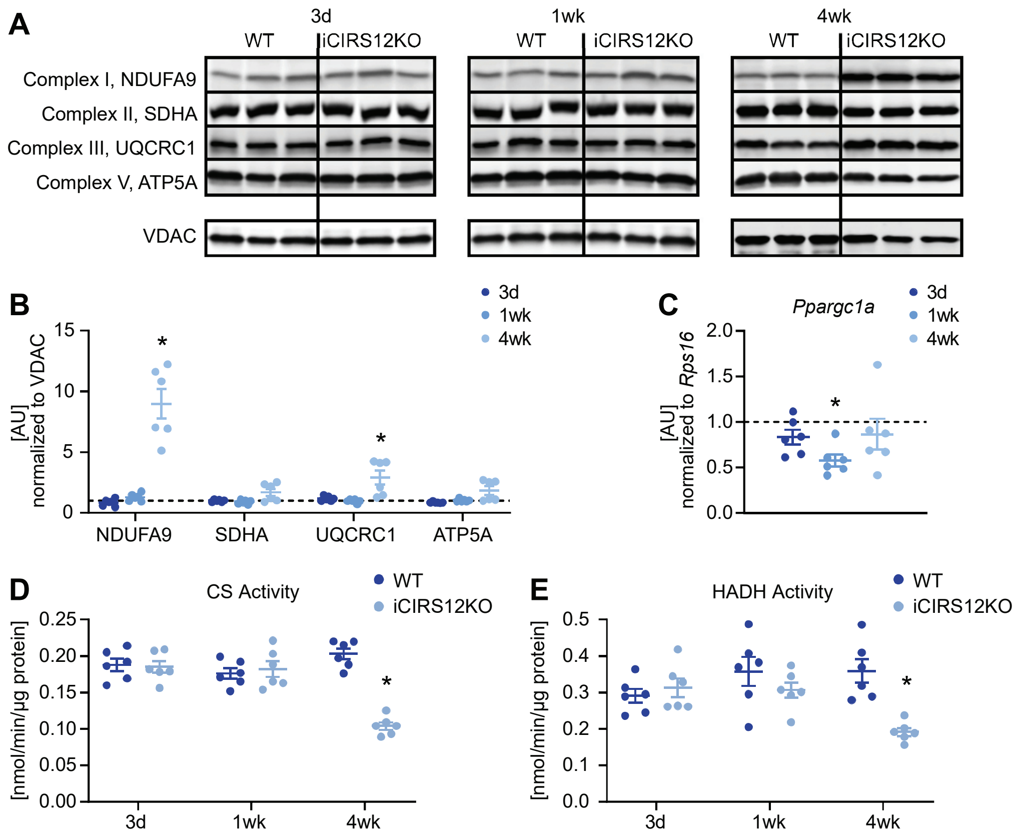

Supplemental Figure 6. *Contractile dysfunction precedes mitochondrial dysfunction iCIRS12KO hearts.*

(A) Representative immunoblots and (B) densitometric analysis of electron transport chain subunits NDUFA9 (complex I), SDHA (complex II), UQCRC1 (complex III), and ATP5A (complex V) normalized to VDAC. Data are presented as fold change relative to WT controls at the same time point (assigned as 1.0; dashed line). (C) mRNA expression of the transcriptional coactivator PGC-1α *(Ppargc1a)* normalized to *Rps16* presented as fold change relative to WT controls at the same time point (assigned as 1.0; dashed line). (D) Citrate synthase (CS) and (E) hydroxyacyl-CoA dehydrogenase (HADH) enzymatic activity. Data are reported as mean values ± SEM, n=6. * p<0.05 vs. WT same time point.

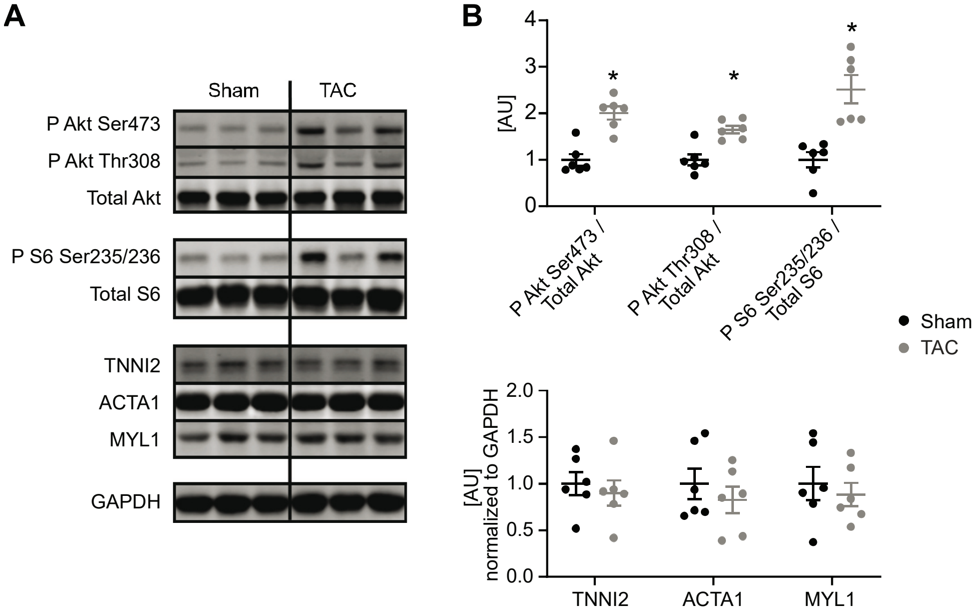

Supplemental Figure 7. *Preserved expression of sarcomeric proteins after four weeks of pressure overload hypertrophy induced by transverse aortic constriction (TAC).*
(A) Representative immunoblots of whole heart extracts from mice four weeks post-TAC surgery and (B) quantification as indicated. TNNI2, troponin I type 2; ACTA1, alpha 1 skeletal muscle actin; MYL1, myosin light chain 1. Data are reported as mean values ± SEM, n=6.* p<0.05 vs. Sham surgery.

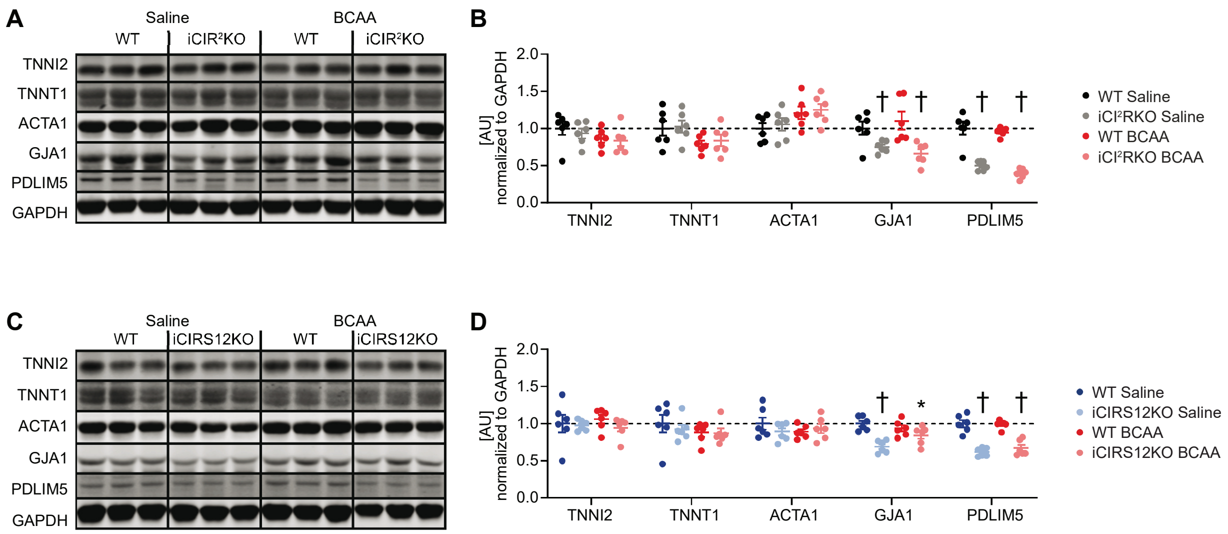

Supplemental Figure 8. *Decreased abundance of cardiac structure proteins in iCIR²KO and iCIRS12KO hearts are not altered by BCAA supplementation (2 wk. week time point).*
Two-way ANOVA was performed to analyze differences by genotype and BCAA treatment, followed by Newman-Keuls post-hoc analysis (# $p<0.05$ for BCAA treatment, $ $p<0.05$ for genotype, & $p<0.05$ for the interaction between BCAA treatment and genotype). (A) Representative immunoblots and (B) quantification of TNNI2, TNNT1 (#), ACTA1 (#), GJA1 ($), and PDLIM5 ($) normalized to GAPDH from ventricle homogenates obtained from iCIR²KO and WT mice after saline or BCAA treatment 2 weeks after gene deletion. Lanes were run on the same gel but were noncontiguous. (C) Representative immunoblots and (D) quantification of TNNI2, TNNT1, ACTA1, GJA1 ($, &), and PDLIM5 ($) normalized to GAPDH from ventricle homogenates obtained from iCIRS12KO and WT mice after saline or BCAA treatment 2 weeks after gene deletion. Lanes were run on the same gel but were noncontiguous. TNNI2, troponin I type 2; TNNT1, troponin T1; ACTA1, alpha 1 skeletal muscle actin; GJA1, gap junction protein alpha 1; PDLIM5, PDZ and LIM domain 5. Data are reported as mean values ± SEM, n=6. * $p<0.05$ vs. Saline same genotype, † $p<0.05$ vs. WT same treatment.

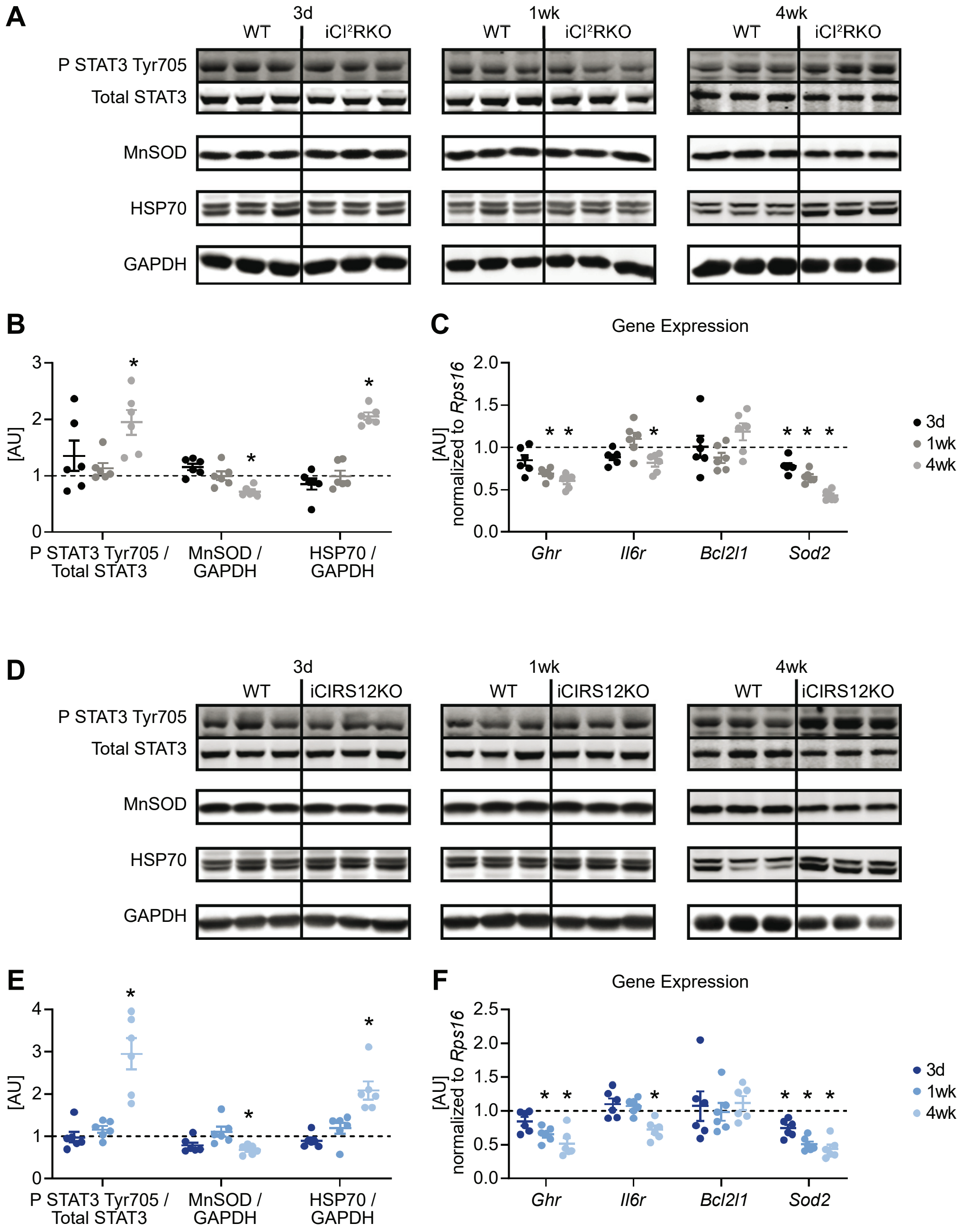

Supplemental Figure 9. *No difference in pro-survival / integrin-mediated signaling in iCIR²KO compared to iCIRS12KO hearts.*

(A) Representative immunoblots, (B) densitometric quantification, and (C) mRNA expression in iCIR²KO hearts as indicated. (D) Representative immunoblots, (E) densitometric quantification, and (F) mRNA expression in iCIRS12KO hearts as indicated. Data are expressed as fold change relative to WT controls at the same time point (assigned as 1.0; dashed line). mRNA expression was normalized to *Rps16*.* $p<0.05$ vs. WT same time point (n=6 each).

Supplemental Tables

Supplemental Table 1. *Contractile function of male iCIR²KO as assessed by transthoracic echocardiography.*

Time Point	Genotype	LVAWt [mm]	Heart Rate [bpm]	LVEDV [µl]	LVESV [µl]	SV [µl]	CO [ml/min]	EF [%]
**pre**	WT	0.85 ± 0.03	564.6 ± 41.6	43.2 ± 6.8	10.7 ± 2.9	32.5 ± 4.1	17.2 ± 1.1	78.3 ± 3.1
	iCIR²KO	0.89 ± 0.04	465.6 ± 31.7	51.6 ± 5.3	14.9 ± 3.1	36.7 ± 3.2	16.5 ± 0.7	73.2 ± 3.9
**3d**	WT	0.95 ± 0.04	708.3 ± 14.0	32.3 ± 3.1	5.5 ± 1.1	26.7 ± 2.5	18.8 ± 1.7	83.2 ± 2.6
	iCIR²KO	0.86 ± 0.03	659.3 ± 17.7 *	37.8 ± 3.1	6.2 ± 1.1	31.6 ± 2.4	20.9 ± 1.8	84.0 ± 2.3
**1wk.**	WT	0.87 ± 0.03	684.6 ± 22.4	34.2 ± 2.1	6.0 ± 0.9	28.2 ± 1.9	19.5 ± 1.7	82.4 ± 2.5
	iCIR²KO	0.81 ± 0.03	571.1 ± 13.0 *	45.5 ± 2.2 *	14.7 ± 0.8 *	30.8 ± 2.3	17.7 ± 1.5	67.2 ± 2.4 *
**2wk.**	WT	0.94 ± 0.04	745.1 ± 14.3	36.1 ± 2.8	5.9 ± 0.9	30.2 ± 2.1	22.5 ± 1.7	84.2 ± 1.9
	iCIR²KO	0.81 ± 0.03 *	589.0 ± 12.7 *	46.0 ± 2.3 *	24.5 ± 2.4 *	21.5 ± 2.2 *	12.5 ± 1.1 *	46.9 ± 4.2 *
**3wk.**	WT	0.88 ± 0.03	703.6 ± 14.0	39.9 ± 1.7	6.4 ± 0.7	33.5 ± 1.3	23.5 ± 1.0	84.1 ± 1.5
	iCIR²KO	0.73 ± 0.02 *	604.5 ± 7.9 *	57.6 ± 5.3 *	35.2 ± 4.7 *	22.5 ± 1.1 *	13.5 ± 0.6 *	40.5 ± 2.9 *
**4wk.**	WT	0.83 ± 0.03	725.8 ± 8.2	39.8 ± 1.5	6.3 ± 1.2	33.6 ± 1.4	24.4 ± 1.1	84.5 ± 2.6
	iCIR²KO	0.65 ± 0.04 *	519.8 ± 74.1 *	63.4 ± 5.2 *	35.8 ± 3.7 *	27.6 ± 2.2 *	14.6 ± 2.3 *	44.1 ± 2.1 *
**6wk.**	WT	0.76 ± 0.04	733.5 ± 9.2	34.5 ± 2.7	4.9 ± 0.6	29.6 ± 2.4	21.7 ± 1.8	85.8 ± 1.5
	iCIR²KO	0.67 ± 0.04	594.4 ± 11.2 *	60.4 ± 7.1 *	34.3 ± 4.9 *	26.1 ± 3.0	15.4 ± 1.8 *	44.2 ± 3.1 *
**12wk.**	WT	0.75 ± 0.03	726.3 ± 10.0	43.9 ± 2.3	4.8 ± 1.1	39.0 ± 2.3	28.4 ± 1.8	89.0 ± 2.2
	iCIR²KO	0.64 ± 0.01 *	599.3 ± 7.1 *	75.0 ± 9.8 *	54.9 ± 10.1 *	20.1 ± 2.0 *	12.1 ± 1.2 *	29.1 ± 3.9 *

Data are reported as mean values ± SEM, n=7-8, * $p<0.05$ vs. WT same time point post gene deletion. LVAWt, left-ventricular anterior wall thickness at diastole; heart rate; LVEDV, left-ventricular end-diastolic volume; LVESV, left-ventricular end-systolic volume; SV, stroke volume; CO, cardiac output; EF, ejection fraction.

Supplemental Table 2. *Contractile function of female iCIR²KO as assessed by transthoracic echocardiography.*

**Time Point**	**Genotype**	**Heart Rate [bpm]**	**LVEDV [µl]**	**LVESV [µl]**	**Ejection fraction [%]**
pre	WT	698.4 ± 12.0	24.6 ± 1.6	2.1 ± 0.2	91.4 ± 0.6
	iCIR²KO	678.2 ± 12.2	24.1 ± 1.6	2.0 ± 0.2	91.5 ± 0.7
1wk.	WT	682.4 ± 12.3	23.9 ± 2.4	3.3 ± 0.6	86.6 ± 1.1
	iCIR²KO	632.4 ± 12.5 *	29.5 ± 3.1	9.4 ± 1.4 *	69.4 ± 2.7 *
4wk.	WT	700.7 ± 5.5	16.7 ± 1.2	2.8 ± 0.3	83.1 ± 1.4
	iCIR²KO	596.7 ± 4.2 *	31.1 ± 2.4 *	13.8 ± 2.6 *	58.1 ± 4.2 *

Data are reported as mean values ± SEM, n=7-11 * $p<0.05$ vs. WT same time point post gene deletion. LVEDV, left-ventricular end-diastolic volume; LVESV, left-ventricular end-systolic volume.

Supplemental Table 3. *Characteristics of iCIR²KO mice.*

Time Point	Genotype	BW [g]	HW [mg]	TL [mm]	HW/BW [mg/g]	HW/TL [mg/mm]	WLW [mg]	WLW / BW [mg/g]	WLW / TL [mg/mm]
**3d**	WT	24.9 ± 1.3	106.7 ± 6.6	16.7 ± 0.2	4.27 ± 0.09	6.37 ± 0.33	149.2 ± 5.5	6.01 ± 0.13	8.93 ± 0.25
	iCIR²KO	24.8 ± 0.9	107.6 ± 4.6	16.5 ± 0.1	4.33 ± 0.07	6.50 ± 0.24	151.1 ± 6.8	6.08 ± 0.14	9.12 ± 0.35
**1wk.**	WT	24.2 ± 1.0	100.5 ± 3.8	16.7 ± 0.1	4.17 ± 0.06	6.00 ± 0.20	153.0 ± 4.5	6.35 ± 0.11	9.14 ± 0.23
	iCIR²KO	25.8 ± 1.0	102.3 ± 3.4	17.0 ± 0.1	3.97 ± 0.10	6.03 ± 0.20	159.9 ± 6.8	6.20 ± 0.18	9.42 ± 0.38
**4wk.**	WT	26.8 ± 1.2	107.8 ± 5.1	17.2 ± 0.1	4.02 ± 0.06	6.26 ± 0.26	167.8 ± 3.5	6.33 ± 0.29	9.76 ± 0.20
	iCIR²KO	26.6 ± 0.5	75.5 ± 2.4 *	17.4 ± 0.2	2.85 ± 0.10 *	4.34 ± 0.13 *	175.9 ± 3.3	6.63 ± 0.14	10.12 ± 0.17

Data are reported as mean values ± SEM, n=6-7, * p<0.05 vs. WT same time point post gene deletion. BW, body weight; HW, heart weight; TL, tibia length; WLW, wet lung weight.

Supplemental Table 4. *Contractile function of iCIRS12KO as assessed by transthoracic echocardiography.*

Time Point	Genotype	LVAWt [mm]	Heart Rate [bpm]	LVEDV [µl]	LVESV [µl]	SV [µl]	CO [ml/min]	EF [%]
**pre**	WT	0.91 ± 0.03	673.6 ± 22.2	32.9 ± 2.7	4.9 ± 0.9	28.0 ± 2.3	18.7 ± 1.6	85.3 ± 2.7
	iCIRS12KO	0.88 ± 0.04	633.4 ± 29.8	40.2 ± 3.0	7.9 ± 1.4	32.2 ± 2.0	20.2 ± 1.4	81.8 ± 2.5
**3d**	WT	0.90 ± 0.04	694.6 ± 19.5	31.7 ± 3.2	4.6 ± 1.0	27.1 ± 2.8	18.7 ± 1.8	85.9 ± 2.2
	iCIRS12KO	0.88 ± 0.04	678.2 ± 19.2	36.4 ± 3.2	5.6 ± 0.9	30.8 ± 2.4	21.0 ± 1.8	85.0 ± 1.1
**1wk.**	WT	0.81 ± 0.04	720.5 ± 15.3	35.6 ± 1.6	5.7 ± 0.8	30.0 ± 1.3	21.6 ± 1.2	84.4 ± 1.9
	iCIRS12KO	0.78 ± 0.04	604.2 ± 12.4 *	45.1 ± 6.0	18.4 ± 2.6 *	26.7 ± 3.8	16.3 ± 2.5	59.7 ± 2.8 *
**2wk.**	WT	0.85 ± 0.05	724.2 ± 5.6	31.1 ± 2.0	5.5 ± 0.6	25.7 ± 1.8	18.6 ± 1.3	82.3 ± 1.6
	iCIRS12KO	0.68 ± 0.04 *	632.7 ± 5.4 *	65.6 ± 7.0 *	44.2 ± 4.4 *	21.4 ± 2.9	13.6 ± 1.9 *	32.4 ± 1.8 *
**3wk.**	WT	0.79 ± 0.01	715.2 ± 5.2	35.9 ± 2.3	6.2 ± 0.8	29.7 ± 1.8	21.2 ± 1.3	83.1 ± 1.8
	iCIRS12KO	0.64 ± 0.02 *	629.4 ± 9.4 *	77.6 ± 8.1 *	57.1 ± 6.3 *	20.5 ± 2.3 *	13.0 ± 1.5 *	27.1 ± 1.8 *
**4wk.**	WT	0.84 ± 0.03	654.4 ± 19.1	34.1 ± 3.0	5.8 ± 1.5	28.3 ± 1.9	18.4 ± 1.2	84.2 ± 2.4
	iCIRS12KO	0.59 ± 0.02 *	633.1 ± 6.1	89.3 ± 10.6 *	66.7 ± 9.6 *	22.6 ± 2.1	14.3 ± 1.3 *	27.3 ± 2.8 *
**6wk.**	WT	0.81 ± 0.03	682.3 ± 17.6	40.0 ± 4.9	6.8 ± 1.0	33.2 ± 4.3	22.2 ± 2.4	82.5 ± 2.0
	iCIRS12KO	0.60 ± 0.04 *	615.6 ± 15.7 *	119.5 ± 12.1 *	96.6 ± 12.3 *	22.9 ± 3.2	14.0 ± 1.8 *	17.7 ± 4.2 *

Data are reported as mean values ± SEM, n=6-13, * $p<0.05$ vs. WT same time point post gene deletion. LVAWt, left-ventricular anterior wall thickness at diastole; heart rate; LVEDV, left-ventricular end-diastolic volume; LVESV, left-ventricular end-systolic volume; SV, stroke volume; CO, cardiac output; EF, ejection fraction.

Supplemental Table 5. *Characteristics of iCIRS12KO mice.*

Time Point	Genotype	BW [g]	HW [mg]	TL [mm]	HW/BW mg/g]	HW/TL [mg/mm]	WLW [mg]	WLW / BW [mg/g]	WLW / TL [mg/mm]
3d	WT	20.6 ± 1.1	100.3 ± 4.5	16.1 ± 0.3	4.89 ± 0.08	6.22 ± 0.25	124.1 ± 3.9	6.09 ± 0.26	7.72 ± 0.28
	iCIRS12KO	22.4 ± 1.1	111.8 ± 6.3	16.3 ± 0.2	5.00 ± 0.15	6.84 ± 0.33	137.5 ± 3.1	6.20 ± 0.29	8.43 ± 0.21
1wk.	WT	22.3 ± 1.3	105.5 ± 5.1	16.1 ± 0.2	4.76 ± 0.14	6.55 ± 0.24	137.2 ± 8.3	6.20 ± 0.36	8.52 ± 0.45
	iCIRS12KO	25.5 ± 0.7	100.1 ± 2.4	16.7 ± 0.2	3.93 ± 0.04 *	5.99 ± 0.10	145.2 ± 5.7	5.69 ± 0.16	8.69 ± 0.31
4wk.	WT	25.7 ± 0.9	113.3 ± 4.8	16.7 ± 0.1	4.42 ± 0.11	6.80 ± 0.28	137.2 ± 6.4	5.36 ± 0.23	8.24 ± 0.39
	iCIRS12KO	24.3 ± 1.1	92.1 ± 5.6 *	16.6 ± 0.1	3.79 ± 0.19 *	5.56 ± 0.32 *	166.4 ± 10.5 *	6.84 ± 0.33 *	10.04 ± 0.61 *

Data are reported as mean values ± SEM, n=6-7, * p<0.05 vs. WT same time point post gene deletion. BW, body weight; HW, heart weight; TL, tibia length; WLW, wet lung weight.

Supplemental Table 6. *Contractile function of iCIR^{2}KO assessed by transthoracic echocardiography following BCAA or Saline treatment.*

Group	Time Point [wk.]	Heart Rate [bpm] #,$	LVEDV [µl] $	LVESV [µl] $	EF [%] $
WT Saline	1 wk.	758.1 ± 10.7	34.8 ± 1.3	4.3 ± 0.7	87.8 ± 1.9
iCIR^{2}KO Saline	1 wk.	652.3 ± 15.2 †	40.0 ± 3.2	6.2 ± 1.1	84.9 ± 1.8
WT BCAA	1 wk.	718.6 ± 16.6	29.7 ± 1.6	3.7 ± 0.6	87.8 ± 1.5
iCIR^{2}KO BCAA	1 wk.	622.5 ± 14.9 †	41.8 ± 3.0 †	9.8 ± 1.1 *†	76.7 ± 2.0 *†
**Group**	**Time Point [wk.]**	**Heart Rate [bpm] $**	**LVEDV [µl] #,$**	**LVESV [µl] $**	**EF [%] $**
WT Saline	2 wk.	743.1 ± 12.9	39.5 ± 1.9	6.3 ± 1.1	84.4 ± 2.6
iCIR^{2}KO Saline	2 wk.	568.0 ± 12.0 †	61.2 ± 4.0 †	25.7 ± 3.7 †	59.4 ± 3.6 †
WT BCAA	2 wk.	742.2 ± 9.8	28.2 ± 2.3	3.9 ± 0.6	86.2 ± 1.2
iCIR^{2}KO BCAA	2 wk.	574.5 ± 11.8 †	52.7 ± 3.5 †	23.4 ± 1.6 †	54.9 ± 2.7 †

Data are reported as mean values ± SEM, n=5-11. Two-way ANOVA was performed to analyze differences by genotype and BCAA treatment, followed by Newman-Keuls post-hoc analysis; $^{\#}$ p<0.05 for BCAA treatment, $ p<0.05 for genotype. LVEDV, left-ventricular end-diastolic volume; LVESV, left-ventricular end-systolic volume; EF, ejection fraction. * p<0.05 vs. Saline same genotype, † p<0.05 vs. WT same treatment.

Supplemental Table 7. *Contractile function of iCIRS12KO assessed by transthoracic echocardiography following BCAA or Saline treatment.*

Group	Time point	Heart Rate [bpm] $	LVEDV [µl] $	LVESV [µl] $	EF [%] $
WT Saline	1 wk.	722.1 ± 17.1	35.0 ± 2.3	4.9 ± 0.6	86.2 ± 1.2
iCIRS12KO Saline	1 wk.	647.7 ± 9.8 †	39.5 ± 3.1	11.3 ± 1.8 †	72.3 ± 2.6 †
WT BCAA	1 wk.	734.5 ± 7.5	31.3 ± 3.1	3.5 ± 0.8	89.5 ± 1.8
iCIRS12KO BCAA	1 wk.	636.1 ± 18.4 †	40.2 ± 2.8	12.6 ± 1.3 †	68.5 ± 2.4 †
**Group**		**Heart Rate [bpm] $**	**LVEDV [µl] $**	**LVESV [µl] $**	**EF [%] $**
WT Saline	2 wk.	733.1 ± 5.9	39.2 ± 2.4	5.9 ± 0.8	84.9 ± 1.8
iCIRS12KO Saline	2 wk.	642.5 ± 8.1 †	61.8 ± 6.0 †	38.6 ± 6.5 †	40.2 ± 6.5 †
WT BCAA	2 wk.	725.8 ± 8.4	39.8 ± 2.5	6.4 ± 1.3	84.5 ± 2.8
iCIRS12KO BCAA	2 wk.	609.9 ± 18.4 †	68.6 ± 3.7 †	41.4 ± 3.0 †	39.7 ± 2.8 †

Data are reported as mean values ± SEM, n=6-8. Two-way ANOVA was performed to analyze differences by genotype and BCAA treatment, followed by Newman-Keuls post-hoc analysis; # $p<0.05$ for BCAA treatment, $ $p<0.05$ for genotype. LVEDV, left-ventricular end-diastolic volume; LVESV, left-ventricular end-systolic volume; EF, ejection fraction. * $p<0.05$ vs. Saline same genotype, † $p<0.05$ vs. WT same treatment.
